# Patterns of seizure frequency reduction in clinical trial participants with lower baseline seizure frequency

**DOI:** 10.1002/epi.70189

**Published:** 2026-03-14

**Authors:** Wesley T. Kerr, Advith S. Reddy, Neo Kok, Katherine N. McFarlane, Lavanya Biju, Jacqueline A. French

**Affiliations:** ^1^ Department of Neurology University of Pittsburgh Pittsburgh Pennsylvania USA; ^2^ Department of Biomedical Informatics and Psychiatry University of Pittsburgh Pittsburgh Pennsylvania USA; ^3^ Comprehensive Epilepsy Center New York University New York New York USA

**Keywords:** antiepileptic drugs, clinical trials, epilepsy, regression to the mean, time to event

## Abstract

**Objective:**

Inclusion and exclusion criteria of clinical trials for seizures aim to select representative participants with a high enough seizure frequency to evaluate the efficacy of treatment in a relatively short double‐blind period. To inform the selection of seizure frequency‐based inclusion criteria, we evaluated the association between baseline seizure frequency and reduction of seizure frequency in the double‐blind period.

**Methods:**

Using data from 11 double‐blind placebo‐controlled trials of antiseizure medications for either focal or generalized onset epilepsy, we evaluated the association of baseline seizure frequency with 50% responder rate and percent reduction of seizure frequency in maintenance. We identified four patterns based on the presence or absence of significant association (*p* < .05) in placebo, active treatment, both, and neither. We also evaluated whether the time to prerandomization monthly seizure count (T‐PSC) design impacted these associations.

**Results:**

In 55% of trials (6/11), there was no significant association of maintenance seizure frequency change with baseline seizure frequency. In 19% of trials (2/11), there were parallel elevations in placebo and active treatment responses for lower baseline seizure frequency. In one trial (1/11), that shift was observed in placebo only, whereas there was a ceiling effect of high response in levetiracetam. In the remaining 19% of trials (2/11), there were more seizure frequency reductions in lower baseline seizure frequencies in active treatment but not placebo. These associations were not modified when the T‐PSC design was used.

**Significance:**

The association of the magnitude of change in seizure frequency with baseline seizure frequency was inconsistent across trials. In eight of 11 trials, these patterns did not reduce the magnitude of difference between active treatment and placebo and thereby may not reduce statistical power. In only one trial did elevated placebo response reduce the difference between active treatment and placebo. In two trials, active treatment appeared more efficacious in lower seizure frequencies.

**Key points**

- The association of baseline seizure frequency with blinded seizure frequency reduction was inconsistent across trials.
- In three of 11 trials, seizure frequency reduction was nonspecifically higher close to the minimum eligibility requirement.
- In one trial, regression to the mean may have reduced statistical power due to a ceiling effect.
- In two of 11 trials, seizure frequency reduction was higher in active treatment only for lower seizure frequencies.
- There were insufficient data to identify a maximum baseline seizure frequency where blinded seizure frequency reductions were smaller.

## 1 INTRODUCTION

Despite the availability of more than 30 antiseizure medications (ASMs), dietary treatment, neuromodulation, and surgical approaches, up to 40% of people with epilepsy continue to have seizures.^1^, ^2^ Especially in medication‐resistant epilepsy, seizures have a profound impact on quality of life, disability, driving privileges, indirect and direct health care costs, and mortality.^3^, ^4^ Therefore, there remains a substantial unmet need to develop and evaluate novel treatments, which require randomized placebo‐controlled trials (RCTs). However, the number of participants recruited per trial site has progressively reduced from more than 10 in the 1990s to fewer than four more recently, which in turn can necessitate use of more than 80 sites to achieve the sample size necessary for statistical power.^5^ Even though the rate of seizure freedom has not substantially changed, we hypothesized that the increased availability of more ASMs, neuromodulatory treatments, and epilepsy surgery may have lowered the proportion of patients with medication‐resistant epilepsy who had sufficiently high seizure frequency (SF) to meet eligibility criteria.^5^, ^6^ Those increasing challenges prompt reevaluation of the historical clinical design of RCTs to promote the evaluation of novel treatments that address the great unmet needs of people with epilepsy.

Changing the eligibility criteria of minimum and maximum SF may have the highest yield in improving recruitment while also maintaining the statistical power of trials. In a study looking at trial eligibility among people with treatment‐resistant focal epilepsy, insufficient SF was the reason for ineligibility in more than 50% of patients.^6^ If that minimum SF could be lowered, then there may be numerous patients who could contribute to the evaluation of novel treatments.

However, it is unknown whether statistical power to observe changes in SF would be lower in patients with lower SF, thereby necessitating either a larger sample size or a longer study.^7^, ^8^ Statistical power may be reduced if the magnitude of the differences in active and placebo arms are reduced and the uncertainty in those differences is higher. Both larger and longer trials would be problematic, because participants on placebo may be exposed to continued consequences of uncontrolled epilepsy, including sudden unexpected death in epilepsy.^4^

Although those concerns are valid, there is increasing evidence that the recruitment challenges may already reduce statistical power through regression to the mean and other factors.^9^, ^10^ The principle of regression to the mean is that a potential participant has a long‐term SF below the minimum eligibility but experiences a temporary worsening of seizures for long enough to become eligible based on retrospective screening and, potentially, the prospective baseline phase. However, once they enter the blinded phase, their SF may naturally reduce to their lower long‐term average, irrespective of blinded treatment.

That nonspecific reduction in seizures is one explanation for the increasing placebo response.^9^ In addition, there has been a concomitant increase in the rate of baseline “failures” (participants who are initially eligible based on retrospective screening SF, but fail to meet SF inclusion criteria during prospective baseline).^5^ In an evaluation restricted to the participants randomized to placebo in 20 RCTs of focal onset seizures, we observed an elevation of placebo response for participants with SF close to that minimum, which provided indirect data‐driven evidence for a potential regression to the mean effect.^11^ In addition, that analysis identified a lower improvement in SF in participants with high baseline SF.^11^ However, that study did not include participants randomized to active treatments. Although this pattern of regression to the mean is one explanation for placebo response in clinical trials, long‐term longitudinal studies of treatment‐resistant epilepsy with and without neuromodulatory treatments also indicate that SF may improve over time.^12^

In this study, we evaluate participants randomized to both active treatment and placebo to understand the association of baseline SF with both placebo and active treatment SF response in multiple RCTs for either focal onset or generalized onset seizures. Statistical power is based upon the magnitude and variability of the difference in SF reduction with active treatment versus placebo.^13^, ^14^ We hypothesized that regression to the mean could either (1) produce parallel shifts in increased response for low SF and thereby maintain statistical power or (2) elevate placebo more than active treatment reductions, thereby reducing the magnitude of difference and, consequentially, statistical power.

## 2 MATERIALS AND METHODS

### 2.1 Trial protocols, registrations, and patient consents

This study addressed these questions by reanalyzing the individual‐participant seizure diaries from 11 placebo‐controlled, double‐blind RCTs for adjunctive pharmaceutical treatments for epilepsy. The individual‐level seizure diary data was provided by each study's sponsor and included the following RCTs indicated by National Clinical Trials number (NCT) and Trial Identifier (Table 1): levetiracetam for primary generalized tonic–clonic seizures in adults and children (NCT00160550, N01507),^15^ brivaracetam for focal onset epilepsy in adults (NCT00464269, N01253; NCT00490035, N01252; NCT00504881, N01254; and NCT01261325, N01358),^16^, ^17^, ^18^, ^19^ lacosamide for focal onset epilepsy in adults (NCT00220415, SP0754; and NCT00136019, SP0755),^20^, ^21^ lamotrigine extended release for focal and generalized onset epilepsy in adults and children (NCT00113165, LAM100034; and NCT00104416, LAM100036),^22^, ^23^ lamotrigine immediate release for generalized onset epilepsy in adults and children (NCT00043901, LAM40097),^24^ and topiramate for generalized onset epilepsy in adults and children (NCT00236704, CR005455).^25^ This analysis focused on deidentified individual‐level participant data and was deemed by the University of Pittsburgh Institutional Review Board to be exempt.

**TABLE 1:** Summary of the included randomized placebo‐controlled trials for medication‐resistant epilepsy with respect to the month and year of the trial start and the group.

ASM	Doses, mg	NCT	Epilepsy type	Response shift pattern	Month/year start
Lacosamide	200, 300 BID	00136019	Focal	None^a^	03/2004
Lacosamide	100, 200 BID	00220415	Focal	None	05/2004
Brivaracetam	2.5, 10, 25 BID	00464269	Focal	None	07/2007
Brivaracetam	Flexible	00504881	Focal & generalized	None	10/1007
Topiramate	200 BID	00236704	Generalized	None	03/1994
Lamotrigine ER	300 daily	00104416	Generalized	None	12/2004
Lamotrigine	150 BID	00043901	Generalized	Parallel	12/2000
Brivaracetam	50, 100 BID	01261325	Focal	Parallel^a^	12/2010
Levetiracetam	1500 BID	00160550	Generalized	Ceiling	09/2001
Brivaracetam	10, 25, 50 BID	00490035	Focal	Active only^a^	02/2009
Lamotrigine ER	300 daily	00113165	Focal	Active only	12/2004

*Note*: Groups reflect observed associations between responder rate or percent reduction in seizure frequency with baseline seizure frequency.

Abbreviations: ASM, antiseizure medication; BID, twice daily; ER, extended release; NCT, ClinicalTrials.gov National Clinical Trial number.

^a^ Difference in the significance of the association with baseline seizure frequency when calculated based on median percent reduction as compared to 50% responder rate (see Table S3).

### 2.2 Calculation of blinded reduction in SF

The primary goal of this study was to evaluate associations of baseline SF with the rate of SF reduction in the double‐blind period, in both the active and placebo arms. We measured SF reduction using the two primary efficacy outcome metrics for clinical trials: percent reduction in SF and 50% responder rate.^26^ The percent reduction in SF was defined for each participant as 100% minus their SF in maintenance divided by their SF in baseline. (The US Food and Drug Administration [FDA] typically includes titration and maintenance in this outcome to address early dropouts, but we chose to focus on the maintenance period only to remove the effect of subtherapeutic ASM doses on the response.) When the percent reduction in SF was greater than or equal to 50%, a patient was considered a 50% responder. The statistical comparison of active treatment to placebo for median percent reduction in SF (MPR) was performed using rank analysis of covariance (ANCOVA) in accordance with the original statistical design of the primary outcome for the FDA.^13^ Analogously, logistic regression was used to compare the 50% responder rate (50RR) in active treatment compared to placebo.^13^ Each trial had differing procedures regarding which confounding factor was included in these regressions. To allow for comparison across trials, we opted to exclude these confounding factors from our analyses. In both regressions, we used mixed effects analyses to account for variability due to country or trial, when that information was available.^27^, ^28^, ^29^

To evaluate if these patterns changed if the time to event design of time to prerandomization monthly seizure count (T‐PSC) was used,^13^, ^26^, ^30^ we performed a sensitivity analysis where the percent reduction in SF and 50% responder rate were calculated before T‐PSC. With the T‐PSC design, each participant's prerandomization baseline 28‐day (monthly) seizure count (PSC) is calculated prior to randomization (e.g., six seizures/month). When the maintenance phase starts, participants continue blinded treatment until they experience at least PSC seizures irrespective of the time needed to observe that number of seizures (e.g., six seizures in 4 weeks); the static end of maintenance (e.g., 12 weeks); or they withdraw for another reason.^13^, ^26^, ^30^ Previously, we showed that the T‐PSC design replicated the primary outcome for all but one of these trials.^13^

### 2.3 Evaluating the association of response with baseline SF

We used locally estimated scatterplot smoothing (LOESS) to visually display the association of response with baseline SF, followed by regression analysis. Due to the nature of SF ranging from one per week to two per day, we evaluated associations with the log of baseline SF.

In a mixed effects multivariable regression, we evaluated the linear association of log SF with response plus the interaction between log SF and randomization arm.^27^, ^28^, ^29^ When evaluating response as defined by RR50, this regression was a mixed effects logistic regression. When evaluating response as defined by MPR, this regression was a mixed effects rank regression. The random effects of these regressions accounted for variability across trials and countries (if available). When a trial had more than one active treatment arm, we grouped all active treatment arms together. In a sensitivity analysis, we also evaluated the patterns when keeping each dose level separate. In another sensitivity analysis, we also considered up to 4th‐order polynomials of log SF.

**FIGURE 1:**
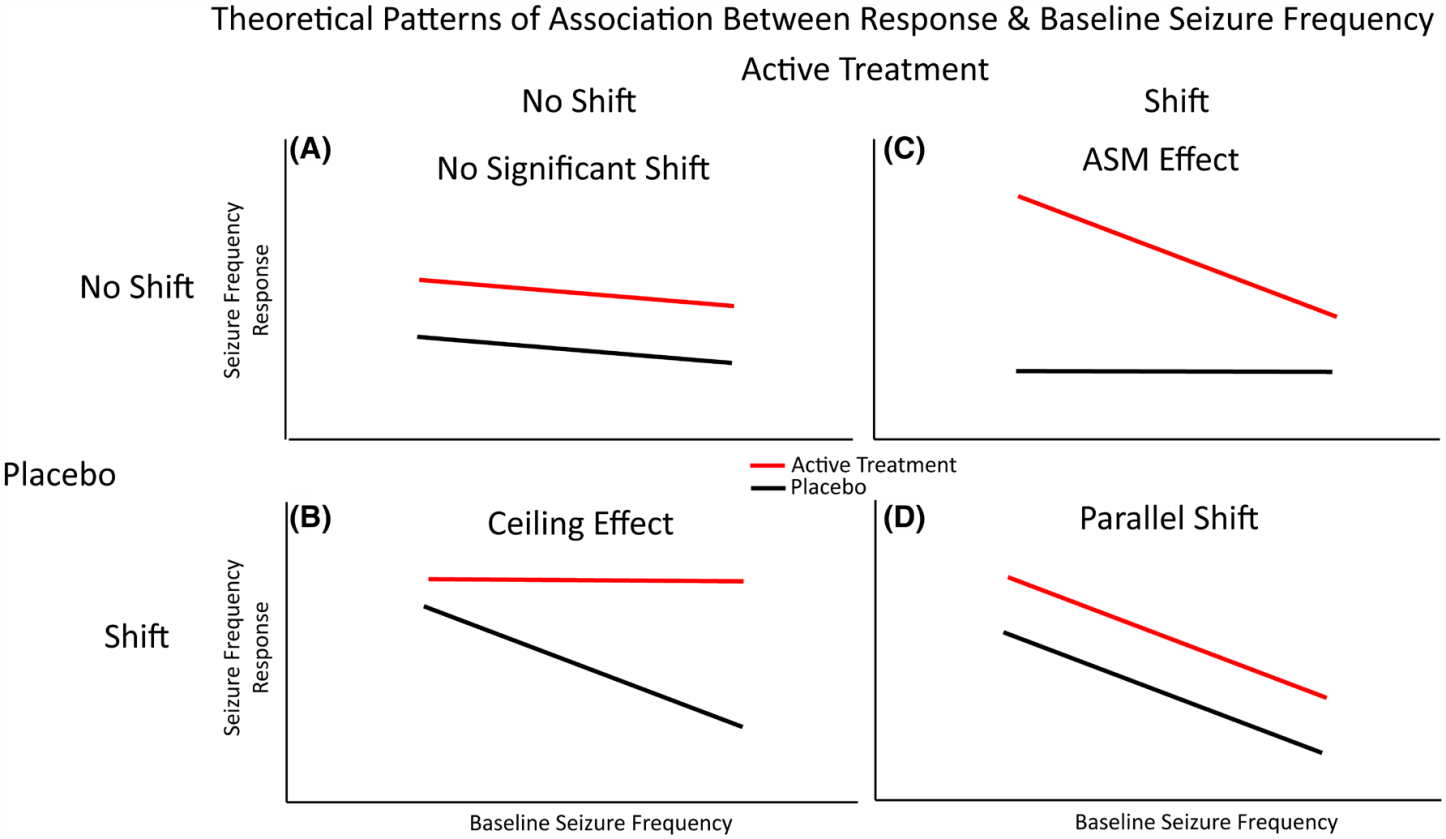
Theoretical patterns of association between seizure frequency response and baseline seizure frequency. Seizure frequency response applies to both percent reduction in seizure frequency and 50% responder rate. ASM, antiseizure medication.

Based on the presence or absence of a significant association of baseline log SF with response in each regression for each trial individually (*p* < .05), we grouped trials into four categories (Figure 1): (1) no association, (2) parallel shift in placebo and active treatment, (3) association in placebo only, and (4) association in active treatment only. If a category had more than one trial, we repeated the mixed effects regressions using an additional random effects term to address heterogeneity across trials. Although this analysis includes 11 trials, we did not correct for multiple comparisons except for with the residual deviance in each multivariable regression so that the magnitude of this association would be clearly apparent for each trial.

We used LOESS to visually display these associations between change in SF and baseline SF. We created LOESS plots for each primary efficacy outcome (MPR and 50RR), all trials combined, each trial individually, each of the four categories of associations, and based on whether the trial was for focal versus generalized onset seizures. LOESS is analogous to a rolling average where complex patterns in the association between variables can be explored without as stringent requirements to define thresholds of SF (e.g., below versus above daily seizures). Instead of thresholds, LOESS uses a span that describes the width of the local rolling average. For each trial, we set the span to .90 log seizures per month, with the exception of the smaller topiramate trial's span of 1 log seizure per month to incorporate sufficient data points to be stable. For the MPR analysis, the LOESS displays the rank of percent reduction in SF, which is analogous to the rank ANCOVA. When results from multiple trials were included in the same visualization, the rank was calculated across all trials and not per trial. (Visualization of the raw or log‐transformed SF was influenced by outliers with marked worsening of SF.) For the 50RR, the uncertainty of the LOESS curve was based on a Clopper–Pearson interval, which used a weighted binomial exact distribution.^31^

## 3 RESULTS

The LOESS plots in Figure 1 illustrate the overall association of RR50 and MPR with baseline SF across all trials for focal onset seizures (Figure 1A,C) and generalized onset seizures (Figure 1B,D).

**FIGURE 2:**
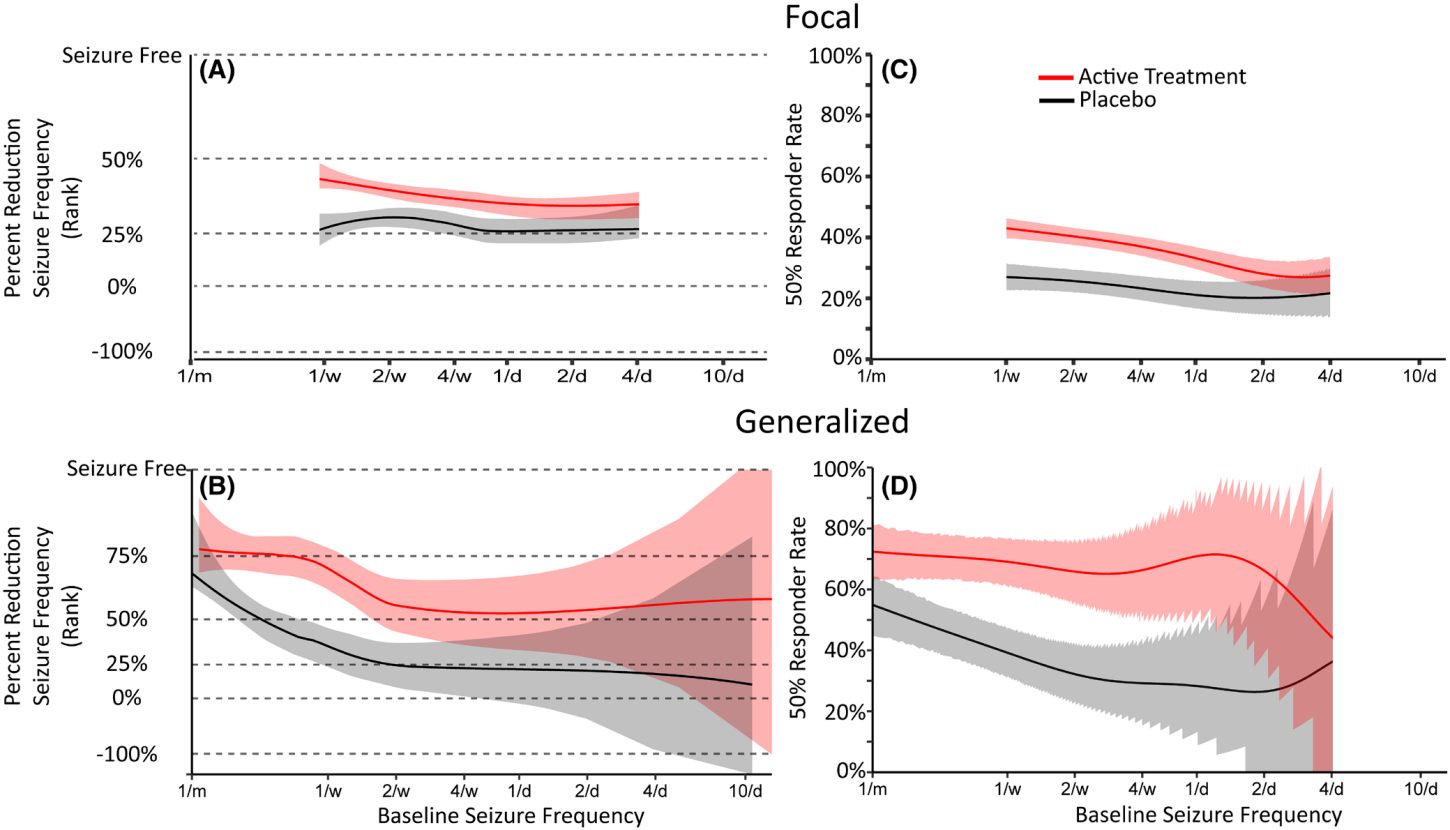
For all focal (A, C) or generalized (B, D) trials combined, the association of baseline seizure frequency with the rank of percent reduction in seizure frequency (A, B) and the 50% responder rate (C, D). Solid lines reflect a weighted average with a span of .9 log seizures per month, whereas shading reflects the local 95% confidence interval. d, day; m, month; w, week.

Table 1 lists the included RCTs and the categories of association of response with baseline log SF: (1) no association, (2) parallel shift in placebo and active treatment, (3) association in placebo but not active treatment, and (4) association in active treatment but not placebo. Figure 2 illustrates these different associations visually for each category. Table S1 describes the results of the meta‐analytic analyses within each category.

In the meta‐analysis across trials with no association within each trial (two lacosamide, topiramate, two of four brivaracetam, one of three lamotrigine),^17^, ^19^, ^20^, ^21^, ^23^ the slope of the association between baseline SF and median/rank percent SF was −4.1 ranks per log SF (SE = 17.8, *p* = .82), with no significant interaction between active treatment and the slope of −33.2 ranks per log SF (SE = 21.5, *p* = .12). The parallel slope of the association with 50RR had log odds ratio of −.15 (SE = .09, *p* = .10) and no significant interaction with active treatment (log odds −.01, SE = .11, *p* = .93).

In the meta‐analysis across trials with parallel shift (lamotrigine for primary generalized tonic–clonic seizures, brivaracetam with 50 and 100 mg twice daily [BID] dosing for focal seizures),^16^, ^24^ the slope of the association between baseline SF and either median/rank percent SF was −20.5 ranks per log SF (SE = 11.5, *p* = .078) or 50RR was log odds −.39 (SE = .12, *p* = .0015). The interaction between active treatment and slope was inconsistent (rank −29.2, SE = 14.9, *p* = .051; log odds −.05, SE = .15, *p* = .76).

In the trial with an association in placebo but not active treatment (levetiracetam for primary generalized tonic–clonic seizures),^15^ the slope of the association between baseline SF and rank percent SF was −22.7 ranks per log SF (SE = 11.6, *p* = .078), with an interaction between active treatment and slope of −29.2 (SE = 14.9, *p* = .051). In the analogous association of 50RR, the slope of the log odds ratio was −1.62 (SE = .43, *p* = .00016) with interaction log odds ratio of 1.31 (SE = .52, *p* = .012).

In the trial with an association in active treatment but not placebo (lamotrigine extended release for focal seizures, brivaracetam for focal seizures with doses of 10, 25, and 50 mg BID),^18^, ^22^ the slope of the association between baseline SF and rank percent SF was −13.4 ranks per log SF (SE = 13.2, *p* = .31), with a log odds ratio of .02 (SE = .17, *p* = .88). The interaction between active treatment and slope was −27.2 ranks per log SF (SE = 16.5, *p* = .10), with a log odds ratio −.76 (SE = .23, *p* = .00081).

Figures S4 through S14 illustrate the LOESS plot for each trial separately. Table S1 describes the mixed effects regression results for each trial. The higher level log‐polynomial regression results did not produce more interpretable findings (Table S2). Each of these patterns was unchanged when trials were reanalyzed using the T‐PSC design (Figures S2–S14).

## 4 DISCUSSION

**FIGURE 3:**
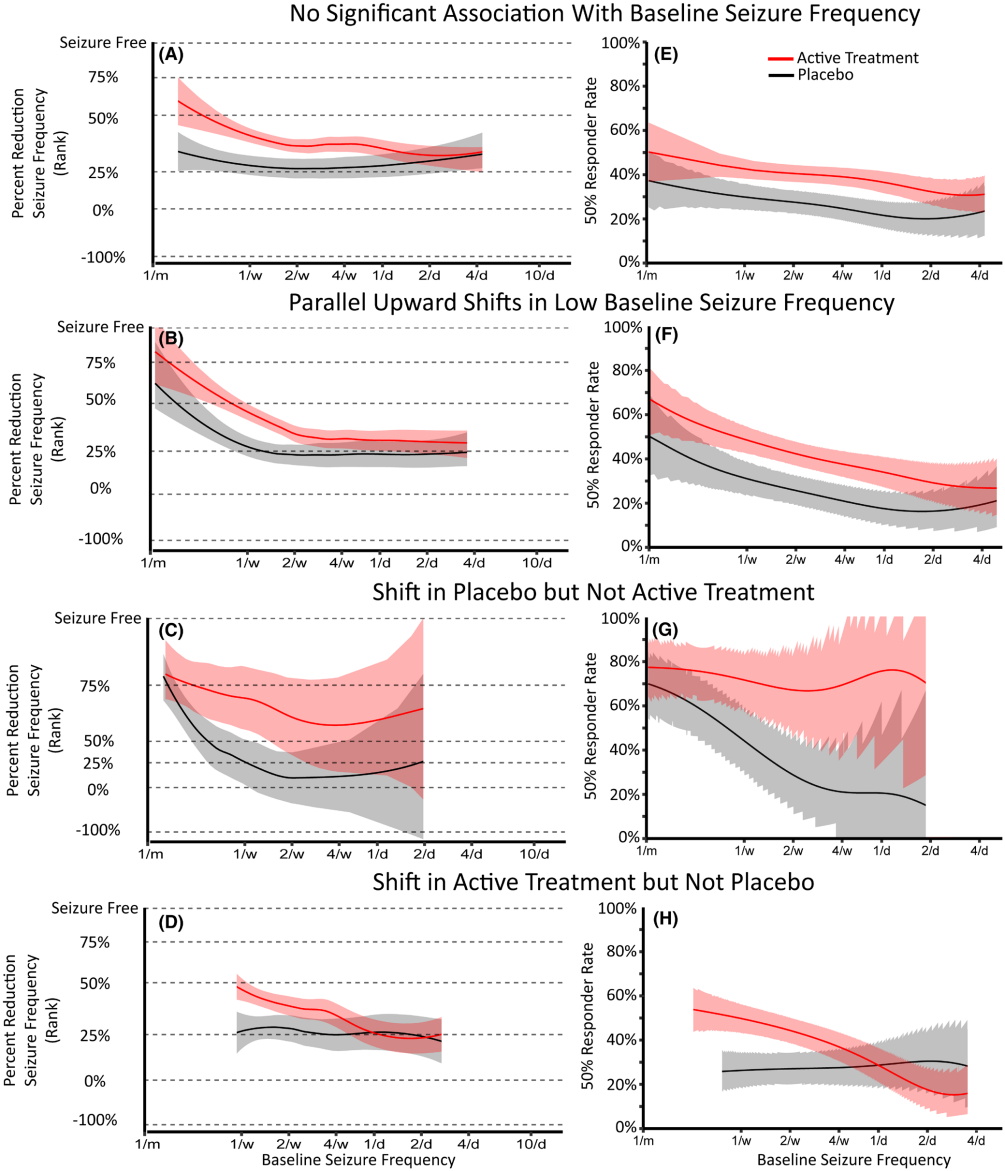
The four patterns of percent reduction in seizure frequency and 50% responder rate associated with baseline seizure frequency: (A, E) no association, (B, F) parallel reduction for lower baseline seizure frequency in both active treatment and placebo, (C, G) reduction for lower baseline seizure frequency in placebo but not in active treatment (ceiling), and (D, H) higher efficacy in lower seizure frequency on active treatment but not placebo. Solid lines reflect a weighted average with a span of .9 log seizures per month, whereas shading reflects the binomial exact 95% confidence interval. See Supplemental Figures for time to prerandomization monthly seizure count results and results of individual trials. d, day; m, month; w, week.

There were inconsistent associations of baseline SF with the reduction in SF in the blinded maintenance phase of clinical trials. The elevation of response for lower baseline SF in three of 11 trials was consistent with the phenomena of regression to the mean (Figure 3B,C,F,G).^9^, ^15^, ^16^, ^24^ For two of those three trials,^16^, ^24^ this phenomenon created parallel shifts in both active treatment and placebo that maintained the difference between treatments and, thereby, may have less impact upon statistical power. However, for the trial of levetiracetam for primary generalized tonic–clonic seizures (Figure 3C,G),^15^ there appeared to be a ceiling effect for active treatment, so the difference between active treatment and placebo was diminished for lower baseline SFs. This leaves two of 11 trials where there appeared to be more improvement in SF for subjects with lower baseline SF (Figure 3D,H).^18^, ^22^ Across all trials, there appeared to be qualitatively less reduction in SF for baseline SFs higher than twice daily seizures, but there were too few participants with that high SF to make conclusions.

These results highlight a potential detrimental impact of the challenge of recruiting patients into the trial on the primary efficacy outcomes of the trial.^5^ Although the effect of regression to the mean can influence all participants irrespective of baseline SF, the impact of eligibility criteria applied to the prospective baseline period can cause a nonspecific elevation in apparent percent SF reduction when baseline SF is close to the minimum requirements.^11^ That occurs because potential participants whose transient worsening in SF was not sustained would be considered screen or baseline failures, the rate of which also has increased over time.^5^ Alternatively, the greater percent reduction in SF for participants with lower baseline SF may reflect greater variability in SF^32^, ^33^, ^34^ or mirror long‐term longitudinal studies showing that SF may improve over time.^12^

In three of 11 trials,^15^, ^16^, ^24^ we observed a significant elevation of treatment response for participants with baseline SF closer to the minimum requirements that would be consistent with regression to the mean. Participant randomization occurred independent of regression to the mean; therefore, this phenomenon would be expected to impact placebo and active treatment equally in most cases. The pattern consistent with regression to the mean also was observed in an analysis of placebo‐only data from 20 trials, some of which also contributed to this analysis.^11^ A similar pattern was observed when the six of 11 trials without significant shift were combined (Figure 3A,E), but that pattern was not statistically significant.^17^, ^19^, ^20^, ^21^, ^23^ In two of three trials,^16^, ^24^ the elevation of response in placebo was paralleled by a concomitant elevation in active treatment, which demonstrates a significant bias in response, but maintains the difference between active treatment and placebo that was needed to demonstrate the efficacy of active treatment. In those eight trials,^17^, ^19^, ^20^, ^21^, ^23^ regression to the mean may have altered the numerical results of median percent reduction and 50RR, but it may not have reduced the statistical power of the trial.

However, in the trial of levetiracetam for primary generalized tonic–clonic seizures, there appeared to be a ceiling effect, where the response to active treatment was so high that regression to the mean may not have been able to further increase response (Figures 3C,G and S4).^15^ Consequentially, the elevated response in placebo but not active treatment led to an underestimation of the efficacy of levetiracetam over placebo and thereby a reduction in statistical power. Although the overall efficacy of levetiracetam remained apparent due to inclusion of participants with higher baseline SF, this reduced estimate of efficacy highlights the potential problems caused by regression to the mean or other similar factors.

Whereas that discussion focuses on the trials with patterns that elevated the placebo response, there were also two trials where no such elevation was observed in placebo (lamotrigine extended release for focal seizures and one brivaracetam trial for focal seizures; Figure 3D,H).^18^, ^22^ Instead, a greater reduction in SF with active treatment was observed in participants with lower baseline SF. That pattern suggested the active treatment could be more efficacious for participants with lower SFs. Longitudinal studies of new onset epilepsy also identified that patients with lower baselines SFs had longer time until they were identified to be treatment‐resistant.^35^ Even though active treatments are evaluated with clinical trials of participants with these relatively high SFs, most participants who receive these treatments after regulatory approval have lower SFs.^6^ Outside of clinical trials, patients with lower baseline SFs were more likely to achieve an at least 1‐year seizure‐free interval with treatment,^36^ but it is unclear whether that reflected a greater percent reduction in SF (e.g., halving of daily seizures would be every other day seizures, but halving of every 6‐month seizures would be yearly seizures). A lower starting SF closer to seizure freedom could be one explanation for why the recent placebo‐controlled trials of cenobamate that recruited participants with at least monthly focal onset seizures had greater rates of seizure freedom, as well as greater percent difference between placebo and active treatment.^37^, ^38^, ^39^ If the observed patterns of higher efficacy in low SFs continued in future trials, then trials that lowered the minimum SF eligibility requirement could observe higher efficacy and thereby have higher statistical power.

Even though regression to the mean did not appear to reduce statistical power except in one trial,^15^ the parallel shifts highlighted the importance of measuring factors associated with placebo response during participant screening, potentially followed by randomization or stratification based on predicted placebo response.^40^, ^41^, ^42^ Another method to address the phenomena of regression to the mean is to lower the minimum SF requirement. That change would increase the overall eligibility rate for clinical trials and thereby ease the pressure of meeting recruitment goals.^6^ Optimistically, that could lower the artificially elevated nonspecific response from regression to the mean and include participants with lower baseline SFs who may experience greater percent SF reductions with active treatment. Because these participants may have greater SF reductions, the overall statistical power could improve.

Cynically, lowering the SF requirement could shift the same problem to lower SF ranges close to the new minimum requirement, as was seen in the trial of levetiracetam for primary generalized tonic–clonic seizures (Figure 3C,G).^15^ Additionally, lowering the SF requirement may require more participants or longer trials to observe sufficient seizures to differentiate placebo and active treatment.^9^ Alternatively, one proposal is to require a minimum SF for retrospective screening but remove that requirement during the prospective baseline phase.^9^ A criticism of that proposal is that prospective seizure diaries are needed to trust and verify retrospective estimates of SF, which can be less accurate.^43^, ^44^

Alternatively, the T‐PSC design may reduce the opportunity for regression to the mean to occur by reducing the time between baseline and maintenance by shortening maintenance in nonresponders.^13^, ^30^ Unfortunately, reanalyses of these trials with the T‐PSC design did not meaningfully change these observed associations. Therefore, the contributors to these patterns may be apparent throughout the maintenance period and were not restricted to the end of the maintenance period.

Although we focus on the influence of participants with low SF, there were also some qualitative patterns in participants with very high SF. Across all the trials except the levetiracetam and perhaps topiramate and one lacosamide trial (Figures S4, S5, and S14),^15^, ^20^, ^25^ the difference between active treatment and placebo appeared smaller above twice daily seizures during baseline (Figure 1). Due to the small number of participants with such high SFs as suggested by the broader confidence bounds in the LOESS plots, we caution against overinterpreting that trend. In our more complex polynomial regressions, there was insufficient evidence to suggest a reduction in response for higher SFs (Table S2). This similar pattern of lower placebo response also was observed in a combined analysis of 20 trials, some of which were included here.^11^

Even if the magnitude of SF reduction was less in participants with very high SF, statistical power should be maintained in these participants. Statistical power is based on effect size, which is related to the magnitude of SF reduction divided by the uncertainty of SF reduction. The higher SF leads to more seizures being observed during each phase of the trial which, in turn, allows for lower uncertainty of SF reduction. Therefore, even if the magnitude of SF was lower in these participants, there may be a commensurate or greater improvement in uncertainty that could compensate for that pattern.^7^, ^10^, ^32^, ^33^, ^45^, ^46^ Detailed evaluation of the complexities of these competing effects is outside the scope of this work.

This approach of reanalyzing a collection of RCTs to identify associations of response rates with baseline SF has important limitations. Although one etiology of these shifts is regression to the mean of SF, high‐quality seizure diaries from before trial recruitment and after trial participation were not available to test that hypothesis.^14^ Each RCT was designed based on the statistical power to differentiate active treatment from placebo, irrespective of baseline SF. Therefore, there was relatively less statistical power for this post hoc analysis. Although mathematically a different version of regression to the mean could occur at very high baseline SFs, the upper limit of eligibility is often so high (e.g., 100 seizures/month) or a countable SF (e.g., absence of status epilepticus) that it does not impact eligibility substantially. We caution against overinterpretation of the qualitative observation that response diminished for high baseline SFs due to the underlying heterogeneity of the combined trials, which evaluated different doses of different medications for different types of epilepsy and were conducted at different times. Additionally, each of these trials relied on human‐reported seizure diaries, which are imperfect.^43^, ^44^ These patterns may not be present when other seizure counting approaches are utilized.^10^, ^44^ To improve the comparability across trials and reduce the potential contribution of subtherapeutic ASM doses, our analyses of percent reduction of SF focused on the maintenance phase only, but the primary FDA efficacy endpoint typically includes both titration and maintenance phases to reduce the potential impact of early dropout.

## 5 CONCLUSIONS

We observed either visually or statistically that participants in many trials with lower baseline SF had more reduction in seizures during the blinded maintenance phase, but those shifts were not expected to reduce statistical power except in one trial. Optimistically in two of 11 trials,^18^, ^22^ that higher efficacy was observed only in active treatment, which may indicate higher efficacy for lower SF. However, in the remaining nine of 11 trials there was also either a visually apparent (*n* = 6) or statistically significant (*n* = 3) shift in placebo and/or active treatment response, which may indicate regression to the mean. In all but one trial,^15^ those shifts may not have reduced statistical power if SF was balanced across placebo and active treatment. In the one exception trial,^15^ regression to the mean may have reduced statistical power to demonstrate that levetiracetam was highly efficacious. Solutions to address these concerning patterns were not immediately clear.

## AUTHOR CONTRIBUTIONS

Wesley T. Kerr developed the original idea for this article, designed the statistical comparisons, modified the figures, and drafted the manuscript. Advith S. Reddy performed analysis of the individual‐level participant data, performed the statistical comparisons, and created figures. Katherine N. McFarlane, Neo Kok, and Lavanya Biju assisted with statistical analysis and interpretation. Jacqueline A. French supervised, assisted in improving interpretability, and provided input at all stages of the project. All authors reviewed manuscript drafts and approved the final version of the work.

## CONFLICT OF INTEREST STATEMENT

No pharmaceutical company contributed to or reviewed this article in any stage of development. W.T.K. received compensation as Associate Editor of *Epilepsia*; writes review articles for *Medlink Neurology*; is a paid consultant for SK Life Sciences, UCB Pharmaceuticals, Jazz Pharmaceuticals, Azurity, Acuta, Ventus, Capsida, Epygenix, Biohaven Pharmaceuticals, the Epilepsy Study Consortium, Cerebral Therapeutics, Neurelis, Noema, EpiTel, QurAlis, Neurona, Neuropace, and Rapport; has collaborative or data use agreements with Eisai, Janssen, Johnson & Johnson, Praxis, Radius Health, and GSK; and has been a site investigator for a trial including UCB Pharmaceuticals and Equilibre Pharmaceuticals. J.A.F. is a paid consultant for Neurelis and Neuropace. J.A.F. receives salary support from the Epilepsy Foundation and for consulting work and/or attending scientific advisory boards on behalf of the Epilepsy Study Consortium for Aeonian/Aeovian, Alterity Therapeutics Limited, Anavex, Arkin Holdings, Angelini Pharma, Arvelle Therapeutics, Athenen Therapeutics/Carnot Pharma, Autifony Therapeutics Limited, Baergic Bio, Biogen, Biohaven Pharmaceuticals, BioMarin Pharmaceutical, BioXcel Therapeutics, Bloom Science, BridgeBio Pharma, Camp4 Therapeutics Corporation, Cerebral Therapeutics, Cerevel, Clinical Education Alliance, Coda Biotherapeutics, Corlieve Therapeutics, Eisai, Eliem Therapeutics, Encoded Therapeutics, Encoded Therapeutics, Engage Therapeutics, Engrail, Epalex, Epihunter, Epiminder, Epitel, Equilibre BioPharmaceuticals, Greenwich Biosciences, Grin Therapeutics, GW Pharma, Janssen Phamaceutica, Jazz Pharmaceuticals, Knopp Biosciences, Lipocine, LivaNova, Longboard Pharmaceuticals, Lundbeck, Marinus, Mend Neuroscience, Marck, NeuCyte, Neumirna Therapeutics, Neurocrine, Neuroelectives USA Corporation, Neuronetics, Neuropace, NxGen Medicine, Ono Pharmaceutical Co., Otsuka Pharmaceutical Development, Ovid Therapeutics, Paladin Labs, Passage Bio, Pfizer, Praxis, Pure Tech, Rafa Laboratories, SK Life Sciences, Sofinnova, Stoke, Supernus, Synergia Medical, Takeda, UCB, Ventus Therapeutics, Xenon, Xeris, Zogenix, Zynerba. J.A.F. also has received research support from the Epilepsy Study Consortium (funded by Andrews Foundation, Eisai, Engage, Lundbeck, Pfizer, SK Life Science, Sunovion, UCB, Vogelstein Foundation), the Epilepsy Study Consortium/Epilepsy Foundation (funded by UCB), GW/FACES, and NINDS. She is on the editorial board of *Lancet Neurology* and *Neurology Today*. She is Chief Medical/Innovation Officer of the Epilepsy Foundation. She has received travel reimbursement related to research, advisory meetings, or presentation of results at scientific meetings from the Epilepsy Study Consortium, the Epilepsy Foundation, Angelini Pharma, Clinical Education Alliance, NeuCyte, Neurocrine, Praxis, and Xenon. The other authors have no conflicts of interest to declare. We confirm that we have read the Journal's position on issues involved in ethical publication and affirm that this report is consistent with those guidelines.

REFERENCES1

Chen
Z
, 
Brodie
MJ
, 
Liew
D
, 
Kwan
P
. Treatment outcomes in patients with newly diagnosed epilepsy treated with established and new antiepileptic drugs: a 30‐year longitudinal cohort study. JAMA Neurol. 2018;75(3):279–286.29279892
10.1001/jamaneurol.2017.3949PMC58858582

Kwan
P
, 
Arzimanoglou
A
, 
Berg
AT
, 
Brodie
MJ
, 
Allen Hauser
W
, 
Mathern
G
, et al. Definition of drug resistant epilepsy: consensus proposal by the ad hoc Task Force of the ILAE Commission on Therapeutic Strategies. Epilepsia. 2010;51(6):1069–1077.19889013
10.1111/j.1528-1167.2009.02397.x3

Harden
C
, 
Tomson
T
, 
Gloss
D
, 
Buchhalter
J
, 
Cross
JH
, 
Donner
E
, et al. Practice guideline summary: sudden unexpected death in epilepsy incidence rates and risk factors: report of the guideline development, dissemination, and implementation subcommittee of the American Academy of Neurology and the American Epilepsy Society. Neurology. 2017;88(17):1674–1680.28438841
10.1212/WNL.00000000000036854

Ryvlin
P
, 
Cucherat
M
, 
Rheims
S
. Risk of sudden unexpected death in epilepsy in patients given adjunctive antiepileptic treatment for refractory seizures: a meta‐analysis of placebo‐controlled randomised trials. Lancet Neurol. 2011;10(11):961–968.21937278
10.1016/S1474-4422(11)70193-45

Kerr
WT
, 
Reddy
AS
, 
Seo
SH
, 
Kok
N
, 
Stacey
WC
, 
Stern
JM
, et al. Increasing challenges to trial recruitment and conduct over time. Epilepsia. 2023;64(10):2625–2634.37440282
10.1111/epi.17716PMC105923786

Kerr
WT
, 
Chen
H
, 
Figuera Losada
M
, 
Cheng
C
, 
Liu
T
, 
French
J
. Reasons for ineligibility for clinical trials of patients with medication resistant epilepsy. Epilepsia. 2023;64(6):e56–e60.36869635
10.1111/epi.175687

Goldenholz
DM
, 
Goldenholz
SR
, 
Moss
R
, 
French
J
, 
Lowenstein
D
, 
Kuzniecky
R
, et al. Does accounting for seizure frequency variability increase clinical trial power?
Epilepsy Res. 2017;137:145–151.28781216
10.1016/j.eplepsyres.2017.07.013PMC56509338

Goldenholz
DM
, 
Tharayil
J
, 
Moss
R
, 
Myers
E
, 
Theodore
WH
. Monte Carlo simulations of randomized clinical trials in epilepsy. Ann Clin Transl Neurol. 2017;4(8):544–552.28812044
10.1002/acn3.426PMC55532269

Goldenholz
DM
, 
Goldenholz
EB
, 
Kaptchuk
TJ
. Quantifying and controlling the impact of regression to the mean on randomized controlled trials in epilepsy. Epilepsia. 2023;64(10):2635–2643.37505116
10.1111/epi.17730PMC1059222710

Goldenholz
DM
, 
Strashny
A
, 
Cook
M
, 
Moss
R
, 
Theodore
WH
. A multi‐dataset time‐reversal approach to clinical trial placebo response and the relationship to natural variability in epilepsy. Seizure. 2017;53:31–36.29102709
10.1016/j.seizure.2017.10.016PMC572266311

Kerr
WT
, 
Suprun
M
, 
Kok
N
, 
Reddy
AS
, 
McFarlane
KN
, 
Kwan
P
, et al. Factors associated with placebo response rate in randomized controlled trials of antiseizure medications for focal epilepsy. Epilepsia. 2025;66(2):407–416.39707877
10.1111/epi.18197PMC1182772012

Potnis
O
, 
Biondo
G
, 
Sukonik
R
, 
Grzeskowiak
C
, 
Cutter
G
, 
Altalib
H
, et al. Seizure frequency trends over time in treatment‐resistant focal epilepsy. JAMA Neurol. 2025;82(12):1257–1264.41114972
10.1001/jamaneurol.2025.4085PMC1268709413

Kerr
WT
, 
Kok
N
, 
Reddy
AS
, 
McFarlane
KN
, 
Stern
JM
, 
Pennell
PB
, et al. Demonstration of group‐level and individual‐level efficacy using time‐to‐event designs for clinical trials of Antiseizure medications. Neurology. 2024;103(4):e209713.39052963
10.1212/WNL.0000000000209713PMC1127139014

Terman
SW
, 
Kirkpatrick
L
, 
Kerr
WT
, 
Akiyama
LF
, 
Baajour
W
, 
Atilgan
D
, et al. Challenges and directions in epilepsy diagnostics and therapeutics: Proceedings of the 17th Epilepsy Therapies and Diagnostics Development conference. Epilepsia. 2024;65(4):846–860.38135921
10.1111/epi.17875PMC1101849515

Berkovic
SF
, 
Knowlton
RC
, 
Leroy
RF
, 
Schiemann
J
, 
Falter
U
, Levetiracetam N01057 Study Group
. Placebo‐controlled study of levetiracetam in idiopathic generalized epilepsy. Neurology. 2007;69(18):1751–1760.17625106
10.1212/01.wnl.0000268699.34614.d316

Klein
P
, 
Schiemann
J
, 
Sperling
MR
, 
Whitesides
J
, 
Liang
W
, 
Stalvey
T
, et al. A randomized, double‐blind, placebo‐controlled, multicenter, parallel‐group study to evaluate the efficacy and safety of adjunctive brivaracetam in adult patients with uncontrolled partial‐onset seizures. Epilepsia. 2015;56(12):1890–1898.26471380
10.1111/epi.1321217

Kwan
P
, 
Trinka
E
, 
van Paesschen
W
, 
Rektor
I
, 
Johnson
ME
, 
Lu
S
. Adjunctive brivaracetam for uncontrolled focal and generalized epilepsies: results of a phase III, double‐blind, randomized, placebo‐controlled, flexible‐dose trial. Epilepsia. 2014;55(1):38–46.24116853
10.1111/epi.1239118

Ryvlin
P
, 
Werhahn
KJ
, 
Blaszczyk
B
, 
Johnson
ME
, 
Lu
S
. Adjunctive brivaracetam in adults with uncontrolled focal epilepsy: results from a double‐blind, randomized, placebo‐controlled trial. Epilepsia. 2014;55(1):47–56.24256083
10.1111/epi.1243219

Biton
V
, 
Berkovic
SF
, 
Abou‐Khalil
B
, 
Sperling
MR
, 
Johnson
ME
, 
Lu
S
. Brivaracetam as adjunctive treatment for uncontrolled partial epilepsy in adults: a phase III randomized, double‐blind, placebo‐controlled trial. Epilepsia. 2014;55(1):57–66.24446953
10.1111/epi.1243320

Halasz
P
, 
Kälviäinen
R
, 
Mazurkiewicz‐Beldzińska
M
, 
Rosenow
F
, 
Doty
P
, 
Hebert
D
, et al. Adjunctive lacosamide for partial‐onset seizures: efficacy and safety results from a randomized controlled trial. Epilepsia. 2009;50(3):443–453.19183227
10.1111/j.1528-1167.2008.01951.x21

Chung
S
, 
Sperling
MR
, 
Biton
V
, 
Krauss
G
, 
Hebert
D
, 
Rudd
GD
, et al. Lacosamide as adjunctive therapy for partial‐onset seizures: a randomized controlled trial. Epilepsia. 2010;51(6):958–967.20132285
10.1111/j.1528-1167.2009.02496.x22

Naritoku
DK
, 
Warnock
CR
, 
Messenheimer
JA
, 
Borgohain
R
, 
Evers
S
, 
Guekht
AB
, et al. Lamotrigine extended‐release as adjunctive therapy for partial seizures. Neurology. 2007;69(16):1610–1618.17938371
10.1212/01.wnl.0000277698.33743.8b23

Biton
V
, 
di Memmo
J
, 
Shukla
R
, 
Lee
YY
, 
Poverennova
I
, 
Demchenko
V
, et al. Adjunctive lamotrigine XR for primary generalized tonic‐clonic seizures in a randomized, placebo‐controlled study. Epilepsy Behav. 2010;19(3):352–358.20937567
10.1016/j.yebeh.2010.07.02224

Biton
V
, 
Sackellares
JC
, 
Vuong
A
, 
Hammer
AE
, 
Barrett
PS
, 
Messenheimer
JA
. Double‐blind, placebo‐controlled study of lamotrigine in primary generalized tonic‐clonic seizures. Neurology. 2005;65(11):1737–1743.16344515
10.1212/01.wnl.0000187118.19221.e425

Biton
V
, 
Montouris
GD
, 
Ritter
F
, 
Riviello
JJ
, 
Reife
R
, 
Lim
P
, et al. A randomized, placebo‐controlled study of topiramate in primary generalized tonic‐clonic seizures. Neurology. 1999;52(7):1330–1337.10227614
10.1212/wnl.52.7.133026

Kerr
WT
, 
Auvin
S
, 
van der Geyten
S
, 
Kenney
C
, 
Novak
G
, 
Fountain
NB
, et al. Time‐to‐event clinical trial designs: existing evidence and remaining concerns. Epilepsia. 2023;64(7):1699–1708.37073881
10.1111/epi.17621PMC1052427927

Yu
Z
, 
Guindani
M
, 
Grieco
SF
, 
Chen
L
, 
Holmes
TC
, 
Xu
X
. Beyond t test and ANOVA: applications of mixed‐effects models for more rigorous statistical analysis in neuroscience research. Neuron. 2022;110(1):21–35.34784504
10.1016/j.neuron.2021.10.030PMC876360028

Tharayil
JJ
, 
Chiang
S
, 
Moss
R
, 
Stern
JM
, 
Theodore
WH
, 
Goldenholz
DM
. A big data approach to the development of mixed‐effects models for seizure count data. Epilepsia. 2017;58(5):835–844.28369781
10.1111/epi.13727PMC542988229

Bates
D
, 
Mächler
M
, 
Bolker
B
, 
Walker
S
. Fitting linear mixed‐effects models using lme4. J Stat Softw. 2015;67(1):1–48.30

French
JA
, 
Gil‐Nagel
A
, 
Malerba
S
, 
Kramer
L
, 
Kumar
D
, 
Bagiella
E
. Time to prerandomization monthly seizure count in perampanel trials: a novel epilepsy endpoint. Neurology. 2015;84(20):2014–2020.25878175
10.1212/WNL.0000000000001585PMC444210131

Clopper
CJ
, 
Pearson
ES
. The use of confidence or fiducial limits illustated in the case of the binomial. Biometrika. 1934;26(4):404–413.32

Zhang
B
, 
Chen
WV
, 
Regalia
G
, 
Goldenholz
DM
, 
Picard
RW
. Statistical characteristics of large‐scale objective tonic‐clonic seizure records from medical smartwatches used in daily life. Epilepsia. 2024;65(11):3255–3264.39287615
10.1111/epi.18109PMC1157364133

Goldenholz
DM
, 
Westover
MB
. Flexible realistic simulation of seizure occurrence recapitulating statistical properties of seizure diaries. Epilepsia. 2022;64(2):396–405.36401798
10.1111/epi.17471PMC990529034

Kerr
WT
, 
Kok
N
, 
McFarlane
KN
, 
Reddy
AS
, 
Biju
L
, 
French
JA
, et al. L‐Relationship between uncertainty and average seizure frequency in clinical trials of antiseizure medications. Epilepsia. 2025. Online ahead of print. 10.1002/epi.70080
PMC130756024147436535

Barnard
SN
, 
Chen
Z
, 
Holmes
M
, 
Kanner
AM
, 
Hegde
M
, 
Kuzniecky
R
, et al. Treatment response to antiseizure medications in people with newly diagnosed focal epilepsy. JAMA Neurol. 2025;82(10):1022–1030.40853673
10.1001/jamaneurol.2025.2949PMC1237912336

Perucca
E
, 
Perucca
P
, 
White
HS
, 
Wirrell
EC
. Drug resistance in epilepsy. Lancet Neurol. 2023;22(8):723–734.37352888
10.1016/S1474-4422(23)00151-537

Chung
SS
, 
French
JA
, 
Kowalski
J
, 
Krauss
GL
, 
Lee
SK
, 
Maciejowski
M
, et al. Randomized phase 2 study of adjunctive cenobamate in patients with uncontrolled focal seizures. Neurology. 2020;94(22):e2311–e2322.32409485
10.1212/WNL.0000000000009530PMC735729338

Krauss
GL
, 
Klein
P
, 
Brandt
C
, 
Lee
SK
, 
Milanov
I
, 
Milovanovic
M
, et al. Safety and efficacy of adjunctive cenobamate (YKP3089) in patients with uncontrolled focal seizures: a multicentre, double‐blind, randomised, placebo‐controlled, dose‐response trial. Lancet Neurol. 2020;19(1):38–48.31734103
10.1016/S1474-4422(19)30399-039

Sperling
MR
, 
Klein
P
, 
Aboumatar
S
, 
Gelfand
M
, 
Halford
JJ
, 
Krauss
GL
, et al. Cenobamate (YKP3089) as adjunctive treatment for uncontrolled focal seizures in a large, phase 3, multicenter, open‐label safety study. Epilepsia. 2020;61(6):1099–1108.32396252
10.1111/epi.16525PMC731755240

Gomeni
R
, 
Bressolle‐Gomeni
F
. Comparison of different machine learning methodologies for predicting the non‐specific treatment response in placebo controlled major depressive disorder clinical trials. Clin Transl Sci. 2025;18(1):e70128.39807769
10.1111/cts.70128PMC1172944441

Gomeni
R
, 
Bressolle‐Gomeni
F
. Model‐informed approach to estimate treatment effect in placebo‐controlled clinical trials using an artificial intelligence‐based propensity weighting methodology to account for non‐specific responses to treatment. J Pharmacokinet Pharmacodyn. 2024;52(1):5.39656323
10.1007/s10928-024-09950-7PMC1163181642

Merlo‐Pich
E
, 
Alexander
RC
, 
Fava
M
, 
Gomeni
R
. A new population‐enrichment strategy to improve efficiency of placebo‐controlled clinical trials of antidepressant drugs. Clin Pharmacol Ther. 2010;88(5):634–642.20861834
10.1038/clpt.2010.15943

Halliday
AJ
, 
Gillinder
L
, 
Lai
A
, 
Seneviratne
U
, 
Fontenot
H
, 
Cameron
T
, et al. The UMPIRE study: a first‐in‐human multicenter trial of bilateral subscalp monitoring for epileptic seizure detection. Epilepsia. 2025;66:3426–3439.40445205
10.1111/epi.18458PMC1245538144

Goldenholz
DM
, 
Tharayil
JJ
, 
Kuzniecky
R
, 
Karoly
P
, 
Theodore
WH
, 
Cook
MJ
. Simulating clinical trials with and without intracranial EEG data. Epilepsia Open. 2017;2(2):156–161.28758158
10.1002/epi4.12038PMC552663945

Romero
J
, 
Larimer
P
, 
Chang
B
, 
Goldenholz
SR
, 
Goldenholz
DM
. Natural variability in seizure frequency: implications for trials and placebo. Epilepsy Res. 2020;162:106306.32172145
10.1016/j.eplepsyres.2020.106306PMC719448646

Goldenholz
DM
, 
Goldenholz
SR
, 
Moss
R
, 
French
J
, 
Lowenstein
D
, 
Kuzniecky
R
, et al. Is seizure frequency variance a predictable quantity?
Ann Clin Transl Neurol. 2018;5(2):201–207.29468180
10.1002/acn3.519PMC5817844

## Supporting information


Table S1.

Figure S1.

## Data Availability

The data that support the findings of this study are available from Vivli. Restrictions apply to the availability of these data, which were used under license for this study. Data are available from http://vivli.org with the permission of Vivli.
