# Supplementary material for: Patterns of seizure frequency reduction in clinical trial participants with lower baseline seizure frequency
Source: Epilepsia. 2026 Mar 14;67(6):2808–19. doi: 10.1002/epi.70189 (PMC13285256; doi:10.1002/epi.70189)
Supplement: Supplementary file 1 — Table S1. [file EPI-67-2808-s002.docx]

|  | Rank Percent Reduction | | | 50% Response Rate | | |
| --- | --- | --- | --- | --- | --- | --- |
| No Significant Shift | Average Rank Shift | SE | p | Log(OR) | SE | p |
| Parallel Slope | -4.1 | 17.8 | 0.82 | -0.15 | 0.09 | 0.10 |
| Difference in ASM Slope | -33.2 | 21.5 | 0.12 | -0.01 | 0.11 | 0.93 |
| Parallel Shift |  |  |  |  |  |  |
| Parallel Slope | -20.5 | 11.6 | 0.078 | -0.39 | 0.12 | 0.0015 |
| Difference in ASM Slope | -29.2 | 14.9 | 0.051 | -0.05 | 0.15 | 0.76 |
| Ceiling Effect |  |  |  |  |  |  |
| Parallel Slope | -22.7 | 5.4 | 0.000050 | -1.62 | 0.43 | 0.00016 |
| Difference in ASM Slope | 13.2 | 7.7 | 0.086 | 1.31 | 0.52 | 0.012 |
| ASM Effect |  |  |  |  |  |  |
| Parallel Slope | -13.4 | 13.2 | 0.31 | 0.02 | 0.17 | 0.88 |
| Difference in ASM Slope | -27.2 | 16.5 | 0.10 | -0.76 | 0.23 | 0.00081 |

Supplemental Table 1 describes the results of the meta-analytic analyses within each category. Parallel slope indicated a shift in both ASM and placebo (negative indicating higher reductions in lower log baseline seizure frequencies), whereas the difference in ASM Slope was the difference in that shift in ASM compared to placebo. Average rank shift indicated the association in the rank regression to evaluate median percent reduction. Abbreviations: antiseizure medication (ASM), standard error (SE), odds ratio (OR).

|  | Rank Percent Reduction | | | 50% Response Rate | | |
| --- | --- | --- | --- | --- | --- | --- |
| No Significant Shift | Average Rank Shift | SE | p | Log(OR) | SE | p |
| Parallel Slope | 991 | 1406 | 0.48 | 0.83 | 7.55 | 0.91 |
| Parallel BSF^2^ | 1615 | 3140 | 0.61 | 22.94 | 17.29 | 0.18 |
| Parallel BSF^3^ | -316 | 4103 | 0.94 | 15.19 | 22.36 | 0.50 |
| Parallel BSF^4^ | -1325 | 2331 | 0.57 | 3.44 | 12.08 | 0.78 |
| Difference in ASM Slope | -2214 | 1514 | 0.14 | -8.79 | 7.97 | 0.27 |
| Difference BSF^2^ | -763 | 3189 | 0.81 | -17.75 | 17.52 | 0.31 |
| Difference BSF^3^ | -401 | 4141 | 0.92 | -18.11 | 22.56 | 0.42 |
| Difference BSF^4^ | 1633 | 2398 | 0.50 | -5.71 | 12.42 | 0.65 |
| Parallel Shift |  |  |  |  |  |  |
| Parallel Slope | -526 | 388 | 0.17 | -10.04 | 3.76 | 0.0076 |
| Parallel BSF^2^ | 1073 | 404 | 0.0081 | 13.17 | 3.79 | 0.0005 |
| Parallel BSF^3^ | -609 | 399 | 0.13 | 0.26 | 3.77 | 0.95 |
| Parallel BSF^4^ | 595 | 398 | 0.14 | 0.86 | 3.90 | 0.83 |
| Difference in ASM Slope | -698 | 496 | 0.16 | -4.55 | 4.75 | 0.34 |
| Difference BSF^2^ | -185 | 507 | 0.72 | -4.26 | 4.77 | 0.37 |
| Difference BSF^3^ | 44 | 506 | 0.93 | -0.84 | 4.80 | 0.86 |
| Difference BSF^4^ | -405 | 503 | 0.42 | 0.04 | 4.82 | 0.99 |
| Ceiling Effect |  |  |  |  |  |  |
| Parallel Slope | -7 | 32 | 0.84 | -150.07 | 159.83 | 0.35 |
| Parallel BSF^2^ | -36 | 32 | 0.26 | -166.96 | 178.76 | 0.35 |
| Parallel BSF^3^ | -7 | 33 | 0.84 | -81.75 | 87.61 | 0.35 |
| Parallel BSF^4^ | -11 | 38 | 0.78 | -22.73 | 24.18 | 0.35 |
| Difference in ASM Slope | -2 | 44 | 0.97 | 152.89 | 159.86 | 0.34 |
| Difference BSF^2^ | -34 | 44 | 0.45 | 165.28 | 178.79 | 0.36 |
| Difference BSF^3^ | 10 | 47 | 0.83 | 84.35 | 87.68 | 0.34 |
| Difference BSF^4^ | 11 | 48 | 0.82 | 24.24 | 24.36 | 0.32 |
| ASM Effect |  |  |  |  |  |  |
| Parallel Slope | -153 | 294 | 0.60 | -1.71 | 5.65 | 0.76 |
| Parallel BSF^2^ | -117 | 316 | 0.71 | -11.06 | 7.37 | 0.13 |
| Parallel BSF^3^ | -199 | 333 | 0.55 | -2.39 | 5.82 | 0.68 |
| Parallel BSF^4^ | -427 | 407 | 0.29 | *-12.27* | *7.25* | *0.09* |
| Difference in ASM Slope | -640 | 367 | *0.081* | -15.32 | 6.70 | 0.022 |
| Difference BSF^2^ | 639 | 382 | 0.10 | 18.48 | 7.97 | 0.020 |
| Difference BSF^3^ | 452 | 393 | 0.25 | 9.69 | 6.78 | 0.15 |
| Difference BSF^4^ | 705 | 452 | 0.12 | 19.27 | 8.11 | 0.017 |

Supplemental Table 2: The higher-level log-polynomial regression results of the association with baseline seizure frequency (BSF) did not produce more interpretable findings. For abbreviations, see Supplemental Table 1.

|  |  | Rank/Median Percent Reduction | | | 50% Response Rate | | |
| --- | --- | --- | --- | --- | --- | --- | --- |
|  |  | **A: No Significant Shift** | | | | | |
| Lacosamide | NCT00136019* | Average Rank | SE | p | Log(OR) | SE | p |
|  | Parallel Slope | 12.7 | 8.2 | 0.12 | 0.02 | 0.21 | 0.94 |
|  | Difference in 200mg BID Slope | -11.1 | 10.5 | 0.29 | 0.08 | 0.25 | 0.75 |
|  | Difference in 300mg BID Slope | -35.1 | 15.4 | 0.023 | -0.22 | 0.35 | 0.53 |
| Lacosamide | NCT00220415 |  |  |  |  |  |  |
|  | Parallel Slope | -15.8 | 10.6 | 0.13 | -0.23 | 0.20 | 0.25 |
|  | Difference in 100mg BID Slope | -0.3 | 13.6 | 0.98 | -0.18 | 0.27 | 0.51 |
|  | Difference in 200mg BID Slope | 21.2 | 14.3 | 0.14 | 0.37 | 0.26 | 0.16 |
| Brivaracetam | NCT00464269 |  |  |  |  |  |  |
|  | Parallel Slope | -13.6 | 11.6 | 0.24 | -0.02 | 0.26 | 0.94 |
|  | Difference in 2.5mg BID Slope | -8.2 | 17.7 | 0.64 | -0.80 | 0.45 | 0.074 |
|  | Difference in 10mg BID Slope | -2.6 | 18.0 | 0.88 | -0.73 | 0.42 | 0.085 |
|  | Difference in 25mg BID Slope | -37.5 | 17.2 | 0.030 | -1.87 | 0.55 | 0.0006 |
| Brivaracetam | NCT00504881 |  |  |  |  |  |  |
|  | Parallel Slope | 14.3 | 10.9 | 0.19 | 0.28 | 0.37 | 0.45 |
|  | Difference in Flexible Dose Slope | -2.1 | 12.3 | 0.86 | 0.11 | 0.41 | 0.78 |
| Topiramate | NCT00236704 |  |  |  |  |  |  |
|  | Parallel Slope | -1.8 | 3.0 | 0.55 | -0.35 | 0.38 | 0.36 |
|  | Difference in 200mg BID Slope | 2.6 | 4.1 | 0.54 | 0.56 | 0.47 | 0.23 |
| Lamotrigine ER | NCT00104416 |  |  |  |  |  |  |
|  | Parallel Slope | -3.1 | 6.3 | 0.62 | 0.02 | 0.36 | 0.95 |
|  | Difference in 300mg daily Slope | -6.3 | 9.5 | 0.51 | -0.14 | 0.59 | 0.82 |

Supplemental Table 3A: Results for each individual trial with no significant shifts. Some p values were less than 0.05, but when combining across all doses they no longer were significant. Abbreviations: twice a day (BID), standard error (SE), extended release (ER), odds ratio (OR).

|  |  | Rank/Median Percent Reduction | | | 50% Response Rate | | |
| --- | --- | --- | --- | --- | --- | --- | --- |
|  |  | **Parallel Shift** | | | | | |
| Lamotrigine | NCT00043901 | Average Rank | SE | p | Log(OR) | SE | p |
|  | Parallel Slope | -8.7 | 4.3 | 0.05 | -0.24 | 0.32 | 0.46 |
|  | Difference in 150mg BID Slope | 2.4 | 6.5 | 0.72 | -0.10 | 0.49 | 0.84 |
| Brivaracetam | NCT01261325* |  |  |  |  |  |  |
|  | Parallel Slope | -1.7 | 12.4 | 0.89 | -0.22 | 0.15 | 0.15 |
|  | Difference in 50mg BID Slope | -18.5 | 17.9 | 0.30 | -0.06 | 0.20 | 0.75 |
|  | Difference in 100mg BID Slope | -47.7 | 18.3 | 0.009 | -0.26 | 0.21 | 0.22 |
|  |  | **Shift in Placebo not ASM** | | | | | |
| Levetiracetam | NCT00160550 | Average Rank | SE | p | Log(OR) | SE | p |
|  | Parallel Slope | -22.7 | 5.4 | 0.000050 | -1.62 | 0.43 | 0.00016 |
|  | Difference in 1500mg BID Slope | 13.2 | 7.7 | 0.086 | 1.31 | 0.52 | 0.012 |
|  |  | **Shift in ASM not Placebo** | | | | | |
| Brivaracetam | NCT00490035* | Average Rank | SE | p | Log(OR) | SE | p |
|  | Parallel Slope | 19.7 | 12.1 | 0.10 | 0.24 | 0.25 | 0.34 |
|  | Difference in 10mg BID Slope | -27.4 | 15.0 | 0.069 | -0.36 | 0.32 | 0.26 |
|  | Difference in 25mg BID Slope | -17.9 | 15.8 | 0.26 | -0.66 | 0.35 | 0.063 |
|  | Difference in 50mg BID Slope | -41.5 | 15.9 | 0.010 | -0.72 | 0.33 | 0.030 |
| Lamotrigine ER | NCT00113165 |  |  |  |  |  |  |
|  | Parallel Slope | -1.4 | 6.7 | 0.83 | 0.10 | 0.24 | 0.67 |
|  | Difference in 300mg daily Slope | -12.2 | 9.1 | 0.18 | -0.63 | 0.33 | 0.054 |

Supplemental Table 3B: Results for each individual trial with significant shifts. Stars, *, align with Supplemental Table 1 where there were differences in conclusions between rank regression of percent reduction and responder rate. The overall groups in Supplemental Table 1 were based on combining across all doses, therefore there are differences between results for individual doses compared to the combination of all doses. Abbreviations: twice a day (BID), standard error (SE), extended release (ER), odds ratio (OR).
