# Supplementary material for: Patterns of seizure frequency reduction in clinical trial participants with lower baseline seizure frequency
Source: Epilepsia. 2026 Mar 14;67(6):2808–19. doi: 10.1002/epi.70189 (PMC13285256; doi:10.1002/epi.70189)
Supplement: Supplementary file 2 — Figure S1. [file EPI-67-2808-s001.docx]

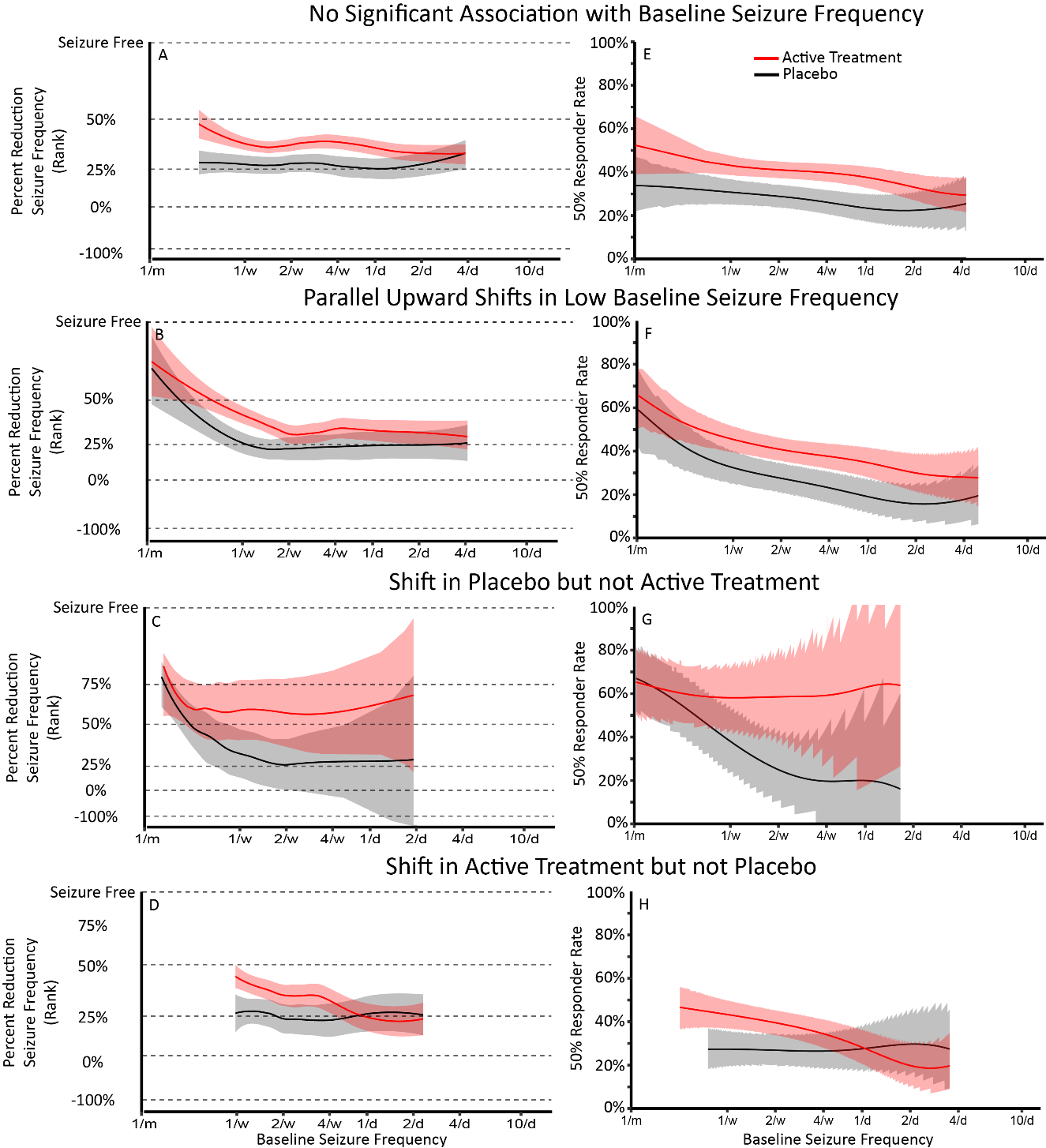


Supplemental Figure 1: This figure is parallel to Figure 3, which used full-length trial data, these outcomes were calculated with the T-PSC design. The four patterns of percent reduction in seizure frequency and 50% responder rate associated with baseline seizure frequency: (A,D) no association, (B,E) parallel reduction for lower baseline seizure frequency in both active treatment and placebo, (C,G) reduction for lower baseline seizure frequency in placebo but not in active treatment (ceiling), and (D,H) higher efficacy in lower seizure frequency on active treatment but not placebo. Solid line reflects a weighted average with a span of 0.9 log seizures per month, whereas shading reflects the binomial exact 95% confidence interval. See Supplemental Figure 1 for results of individual trials.


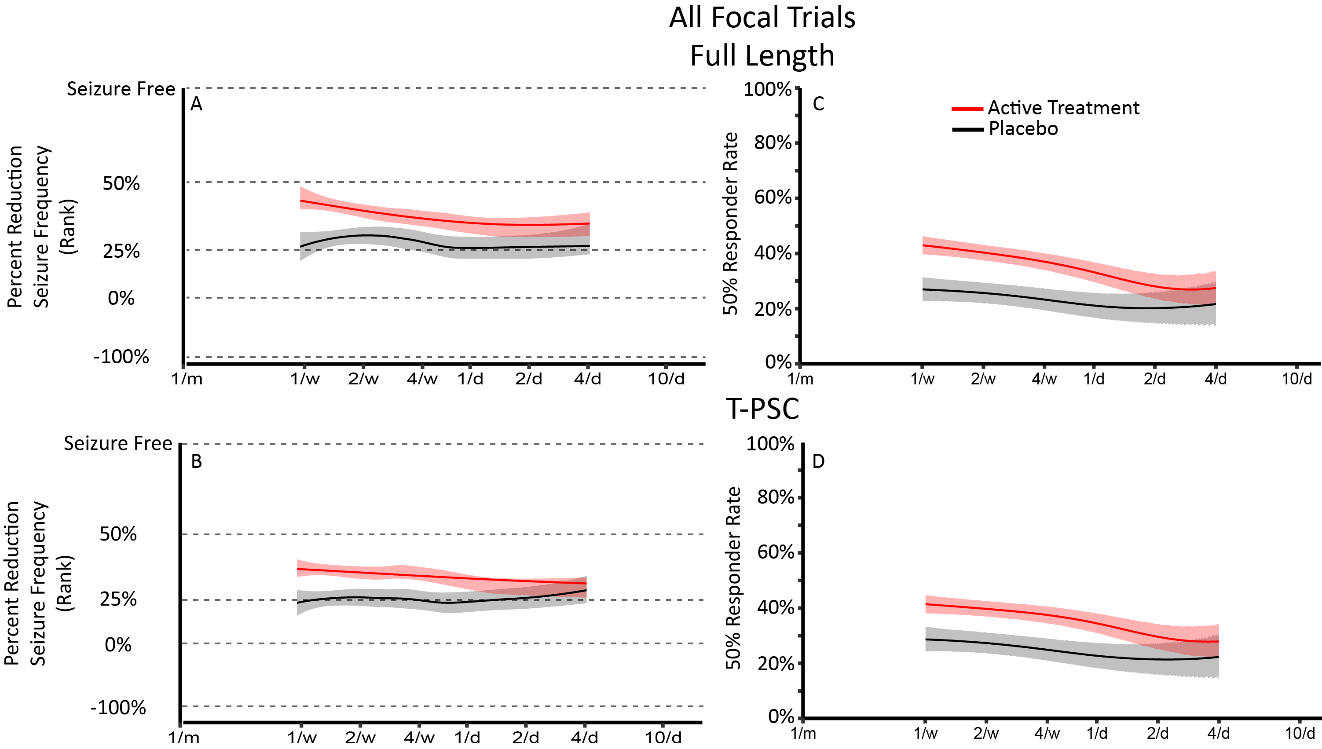


Supplemental Figure 2: Comparison of these patterns of percent reduction in seizure frequency and 50% responder rate when calculated with full length maintenance diaries (A, C) as compared to the T-PSC design (B, D) for focal-onset seizure trials. There was no significant change in these patterns when the outcome was calculated with the T-PSC design. Error bars of percent reduction of seizure frequency reflect 95% confidence intervals of the rank. Error bars of 50% responder rate reflect Clopper-Pearson 95% confidence intervals.


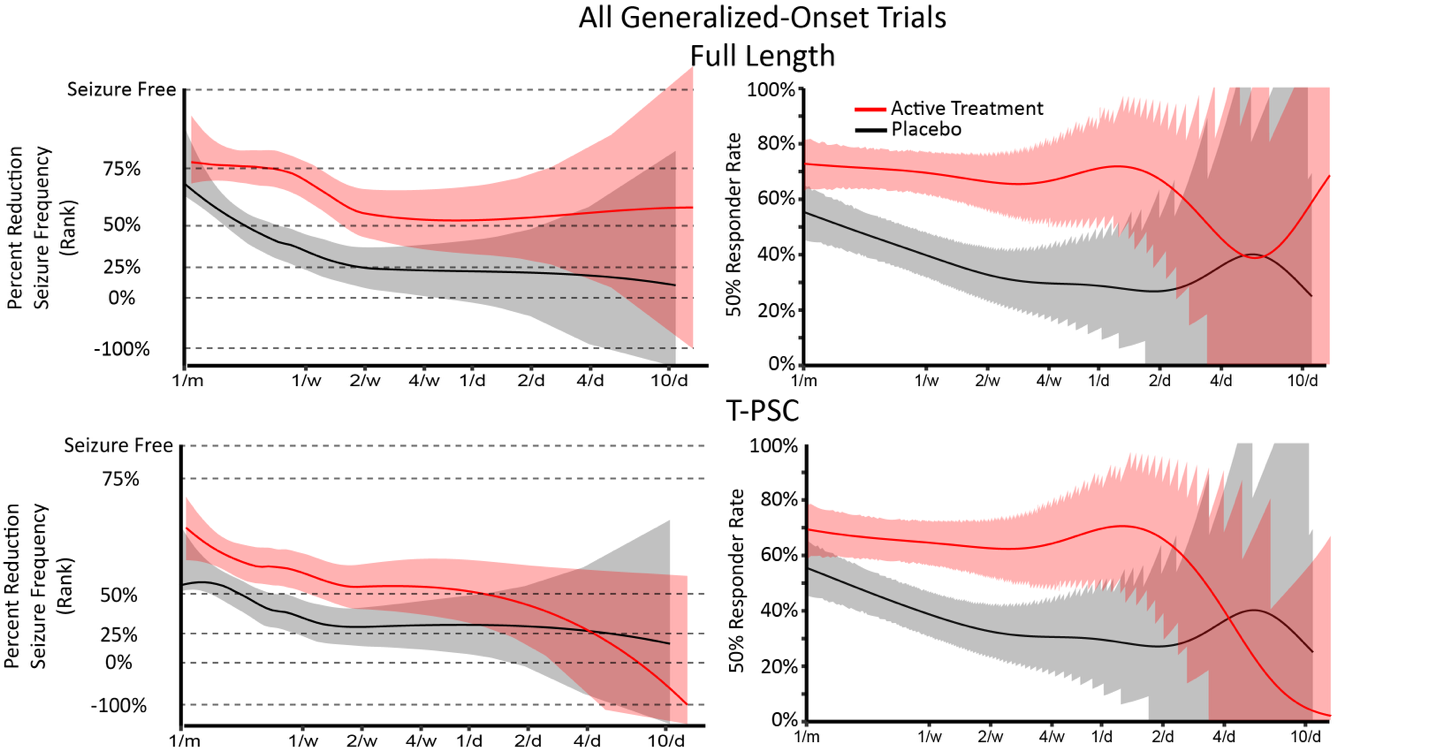


Supplemental Figure 3: Comparison of these patterns of percent reduction in seizure frequency and 50% responder rate when calculated with full length maintenance diaries (A, C) as compared to the T-PSC design (B, D) for generalized-onset seizure trials. There was no significant change in these patterns when the outcome was calculated with the T-PSC design. Error bars of percent reduction of seizure frequency reflect 95% confidence intervals of the rank. Error bars of 50% responder rate reflect Clopper-Pearson 95% confidence intervals.


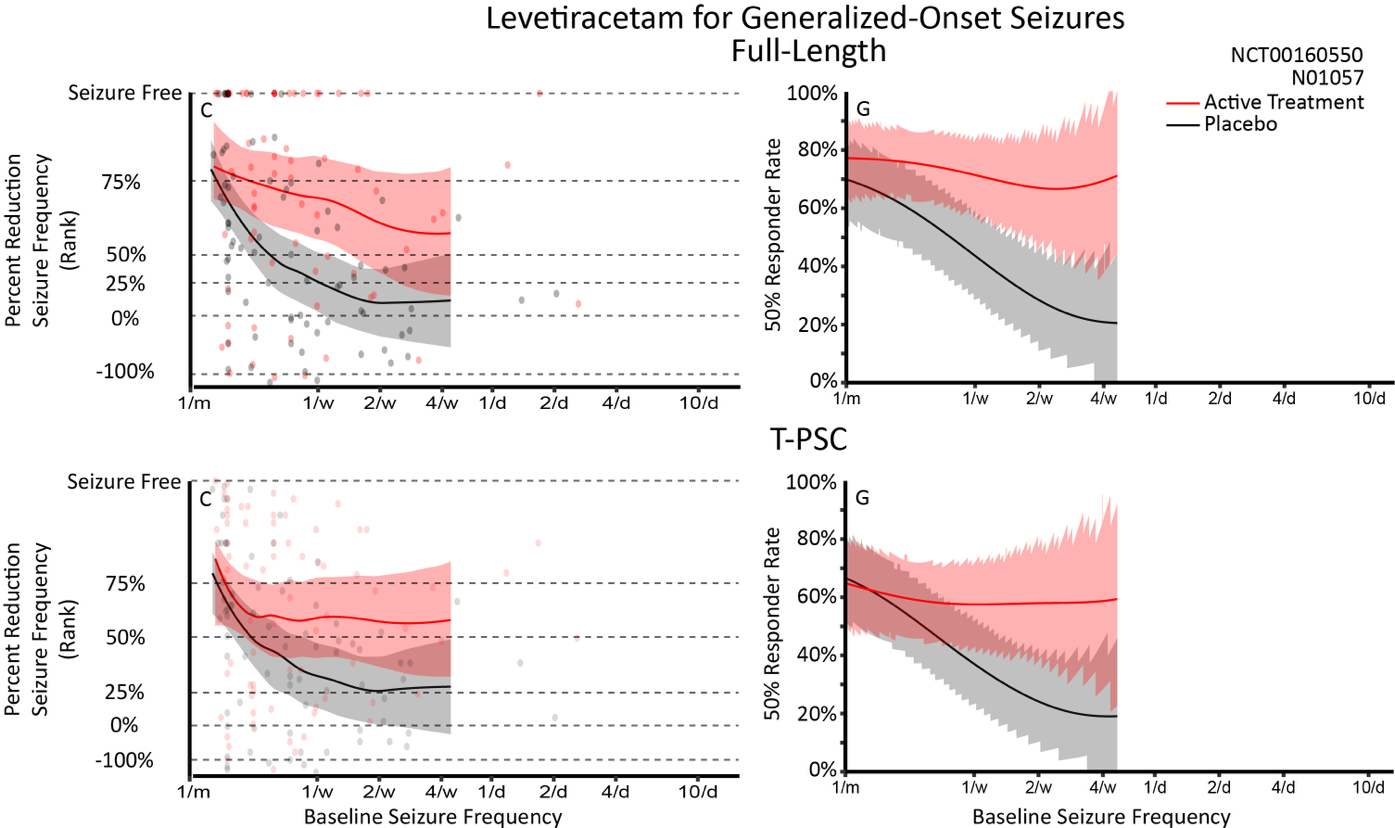


Supplemental Figure 4: Illustration of the individual results from the trial of levetiracetam for primary generalized tonic-clonic seizures (N01057, NCT00160550). Ceiling effect: significant shift in increased percent reduction and 50% reduction in people with lower seizure frequency for placebo (parallel slope MPR p=0.000050; 50RR p=0.00016), but no significant shift in active treatment (difference in slope adds to 0, MPR p=0.086; 50RR p=0.012). Each dot reflects an individual participant. Error bars of percent reduction of seizure frequency reflect 95% confidence intervals of the rank. Error bars of 50% responder rate reflect Clopper-Pearson 95% confidence intervals.


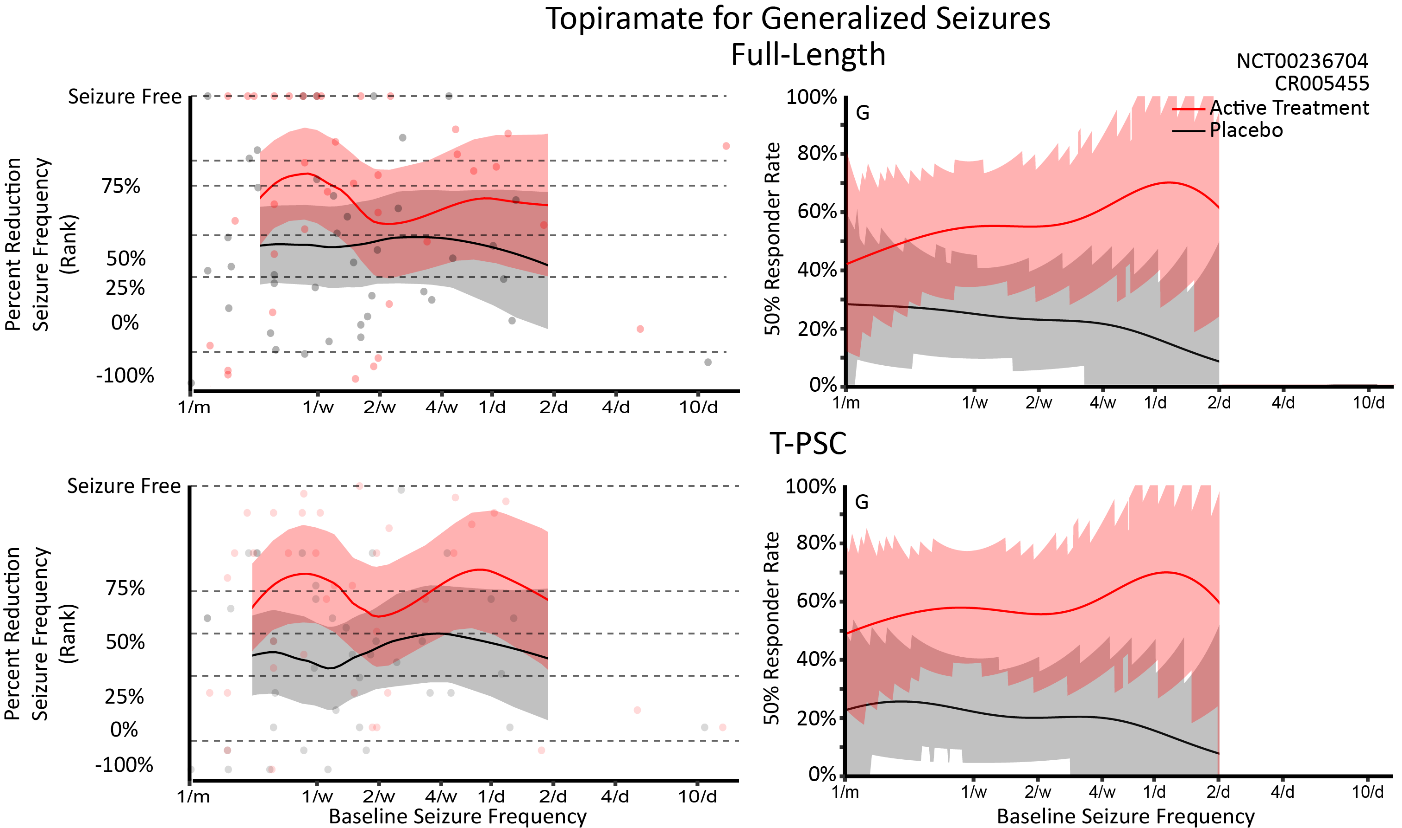


Supplemental Figure 5: Illustration of the individual results from the trial of topiramate for primary generalized tonic-clonic seizures (CR005455, NCT00236704). No significant shift in either percent reduction or 50% responder rate (MPR p=0.55; 50RR p=0.036). Each dot reflects an individual participant. Each dot reflects an individual participant. Error bars of percent reduction of seizure frequency reflect 95% confidence intervals of the rank. Error bars of 50% responder rate reflect Clopper-Pearson 95% confidence intervals.


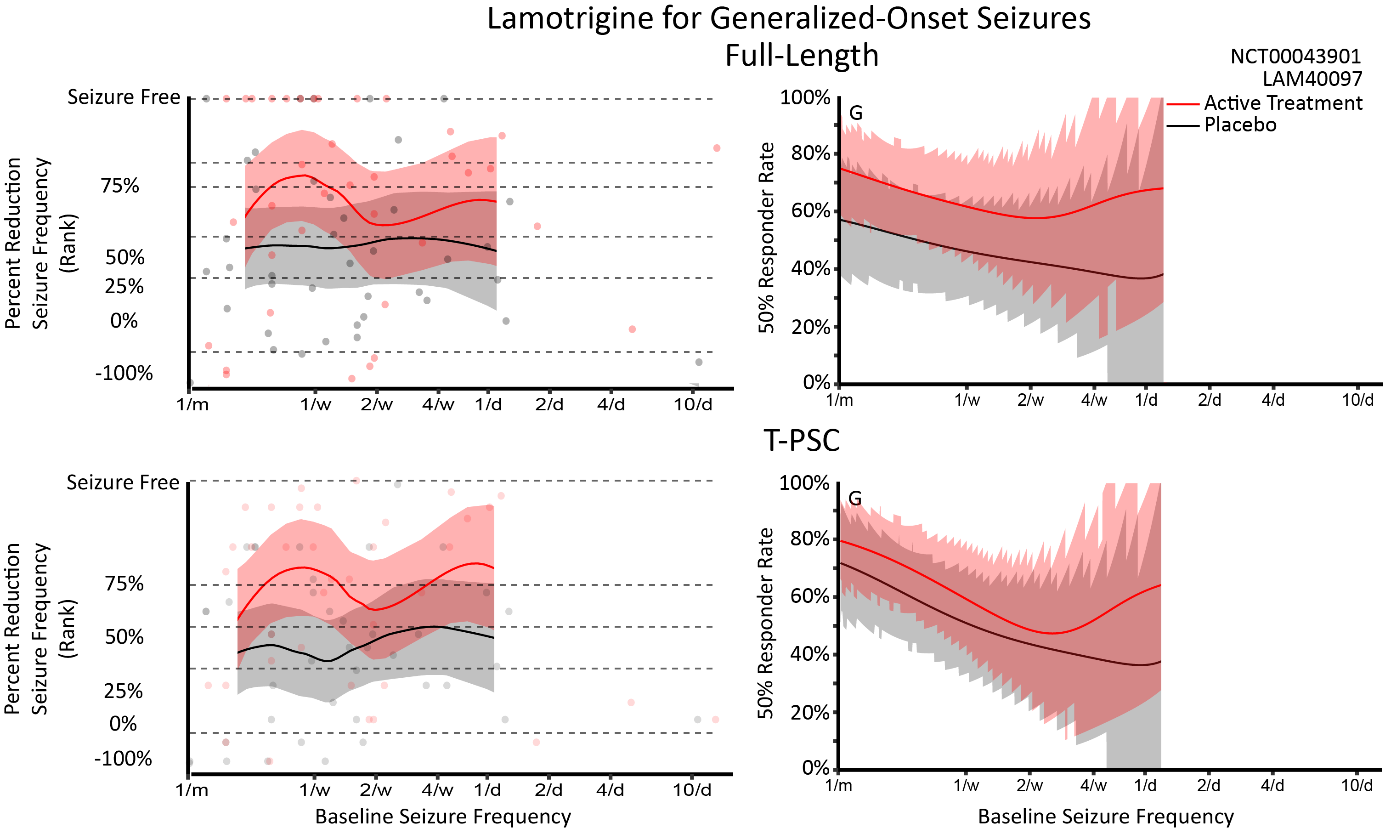


Supplemental Figure 6: Illustration of the individual results from the trial of lamotrigine for primary generalized tonic-clonic seizures (LAM40097, NCT00043901). Significant parallel shift in increased percent reduction in people with lower seizure frequency (MPR p=0.05) but that parallel shift was not significant for 50% responder rate (50RR p=0.46). Each dot reflects an individual participant. Error bars of percent reduction of seizure frequency reflect 95% confidence intervals of the rank. Error bars of 50% responder rate reflect Clopper-Pearson 95% confidence intervals.


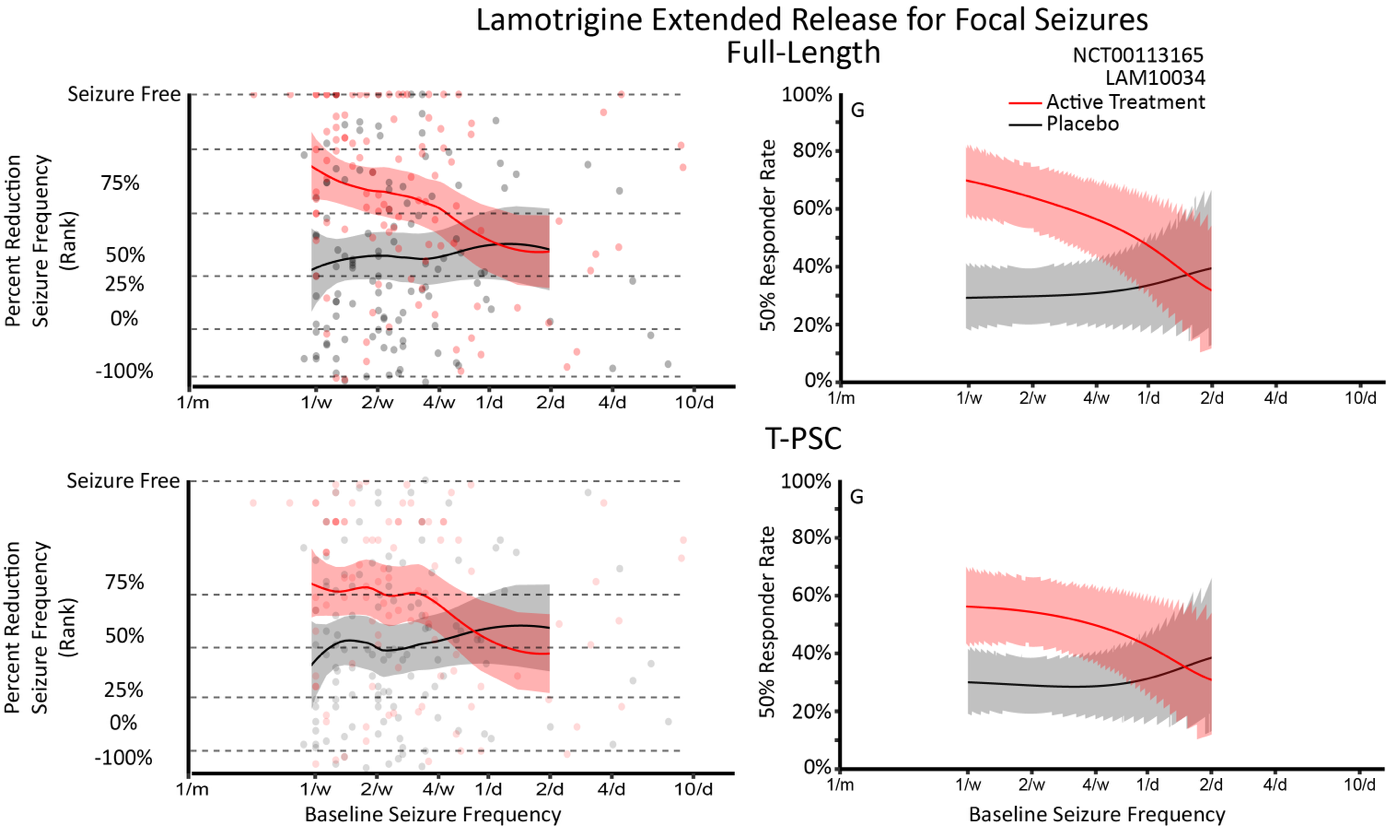


Supplemental Figure 7: Illustration of the individual results from the trial of lamotrigine extended release for focal seizures (LAM40097, NCT00113165). Significant shift in increased percent reduction and 50% reduction in people with lower seizure frequency in active treatment (difference in ASM slope MPR p=0.18; 50RR p=0.054), but not placebo (MPR p=0.83, 50RR p=0.67). Each dot reflects an individual participant. Error bars of percent reduction of seizure frequency reflect 95% confidence intervals of the rank. Error bars of 50% responder rate reflect Clopper-Pearson 95% confidence intervals.


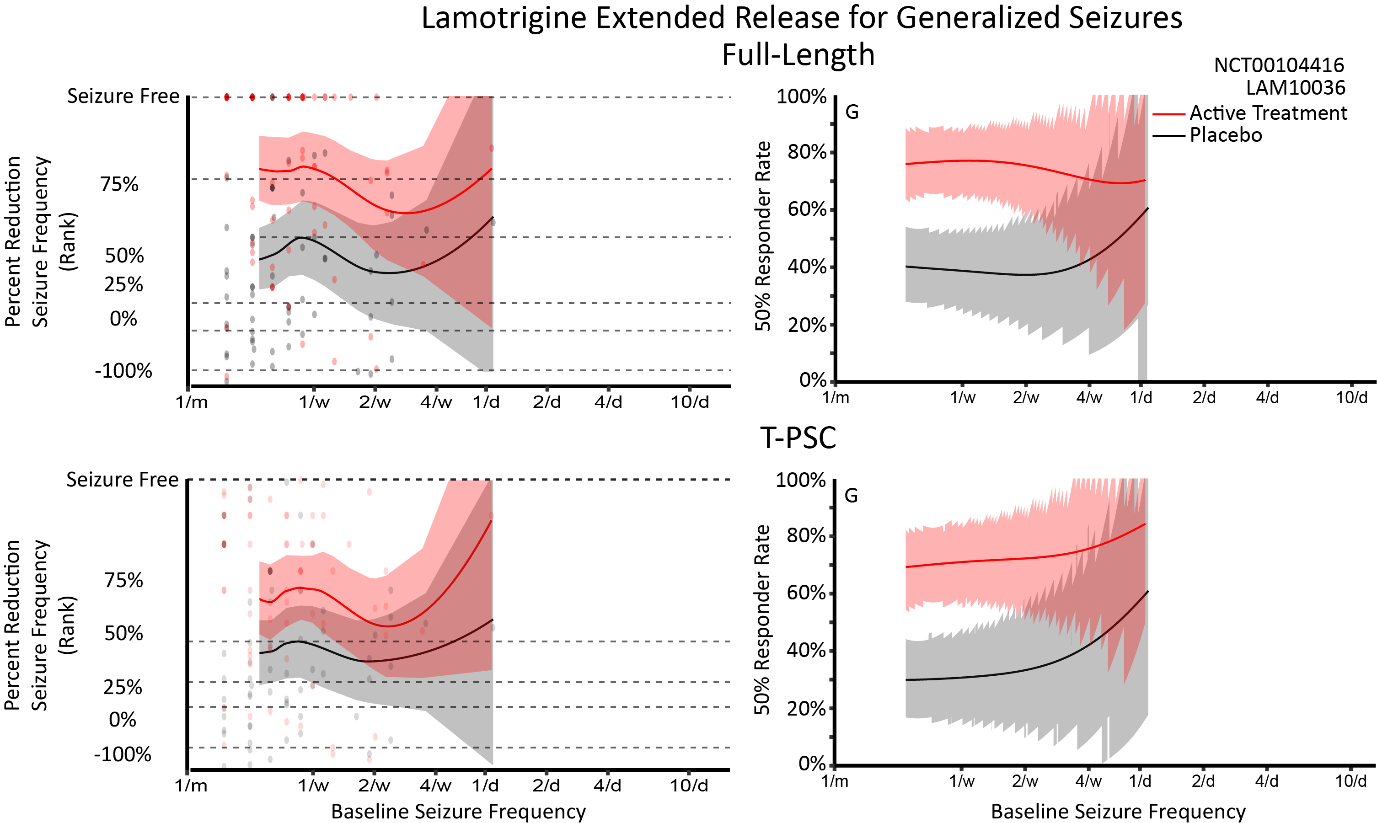


Supplemental Figure 8: Illustration of the individual results from the trial of lamotrigine extended release for primary generalized tonic-clonic seizures (LAM100036, NCT00104416). No significant shift in either percent reduction or 50% responder rate (MPR p=0.062, 50RR p=0.95). Each dot reflects an individual participant. Error bars of percent reduction of seizure frequency reflect 95% confidence intervals of the rank. Error bars of 50% responder rate reflect Clopper-Pearson 95% confidence intervals.


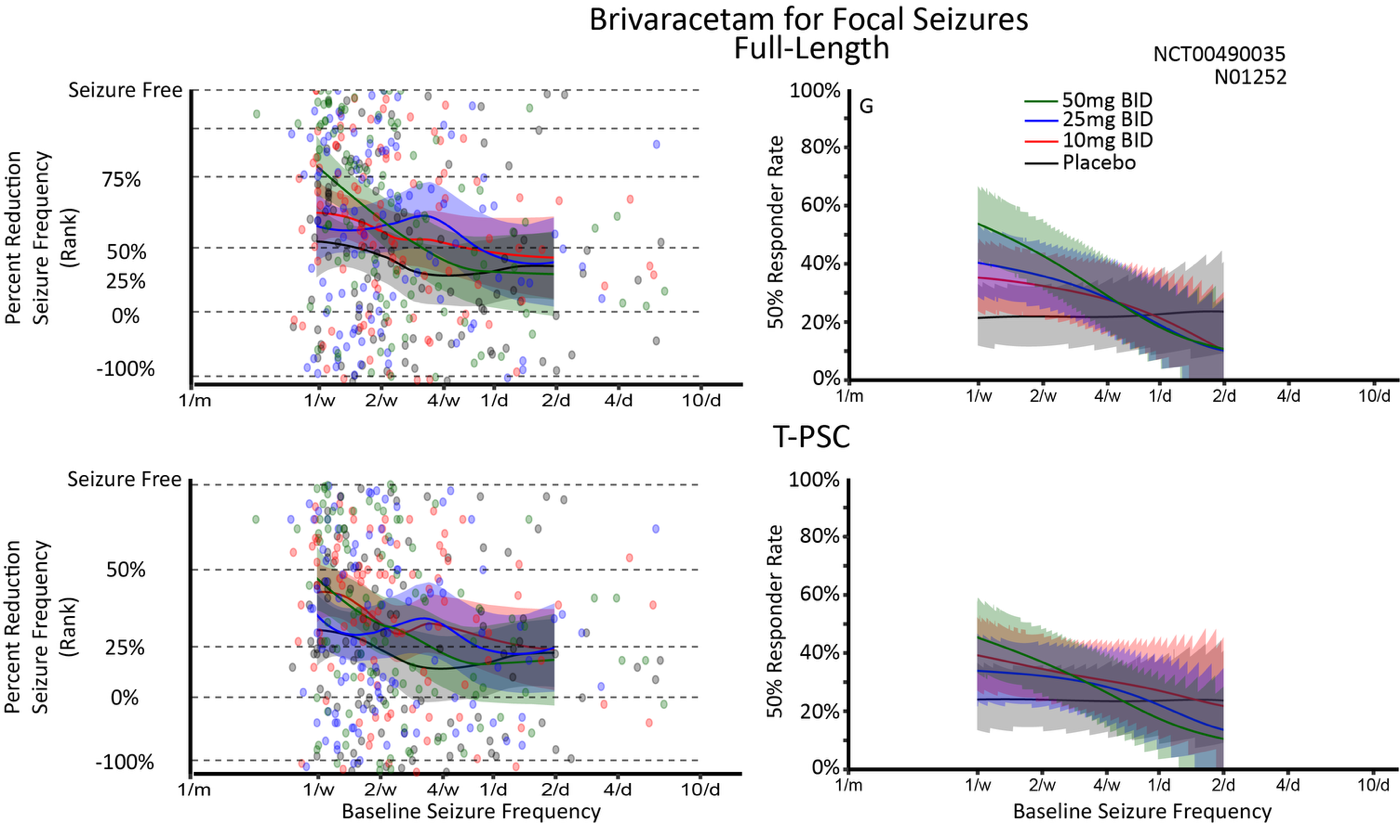


Supplemental Figure 9: Illustration of the individual results from the trial of brivaracetam for focal seizures (N01252, NCT00490035). Significant shift in increased percent reduction and 50% reduction in people with lower seizure frequency for active treatment (difference in ASM slope 50mg BID MPR p=0.010/50RR p=0.030; 25mg BID p=0.26/50RR p=0.063; 10mg BID MPR p=0.069/50RR p=0.26), but not placebo (parallel slope MPR p=0.10; 50RR p=0.34). Each dot reflects an individual participant. Error bars of percent reduction of seizure frequency reflect 95% confidence intervals of the rank. Error bars of 50% responder rate reflect Clopper-Pearson 95% confidence intervals.


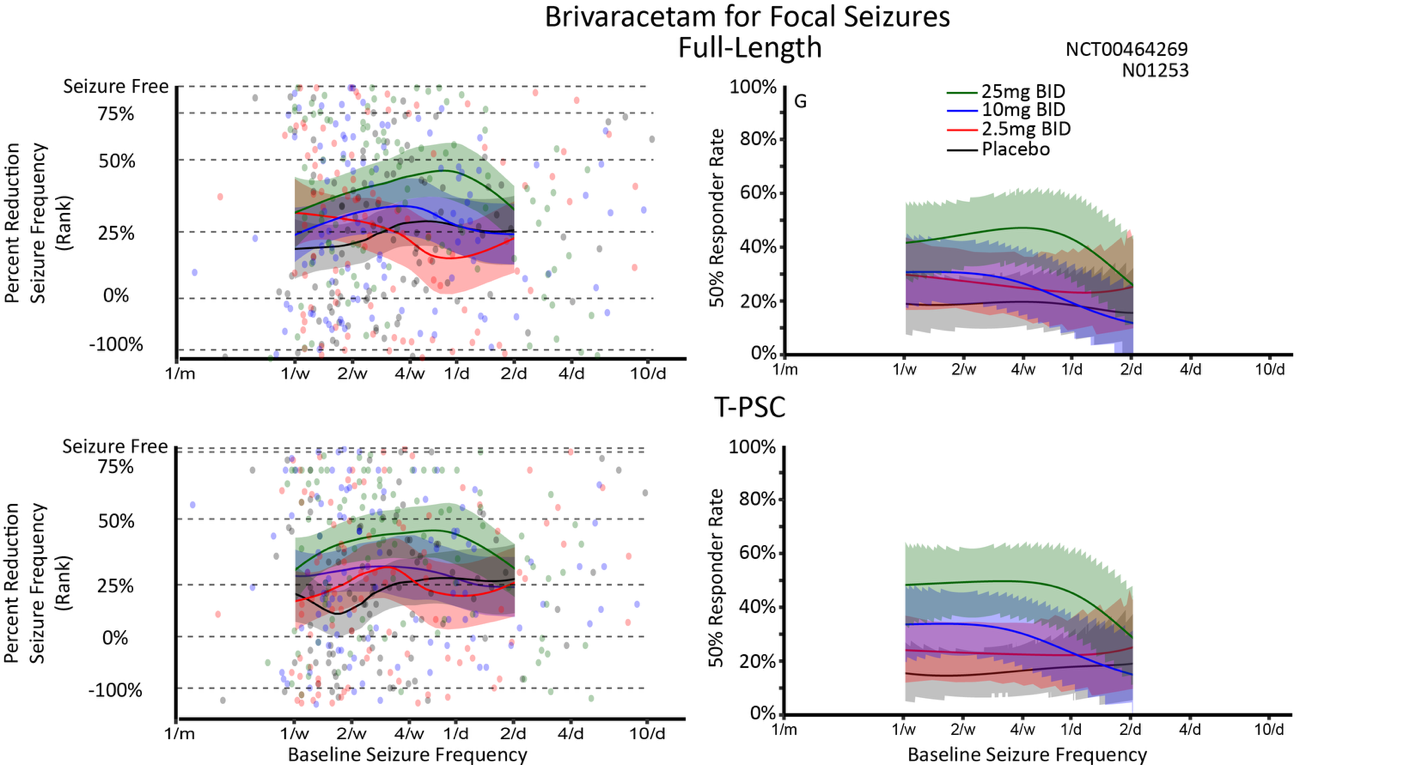


Supplemental Figure 10: Illustration of the individual results from the trial of brivaracetam for focal seizures (N01253, NCT00464269). No significant shift in either percent reduction or 50% responder rate (parallel slope MPR p=0.24; 50RR p=0.94). Each dot reflects an individual participant. Error bars of percent reduction of seizure frequency reflect 95% confidence intervals of the rank. Error bars of 50% responder rate reflect Clopper-Pearson 95% confidence intervals.


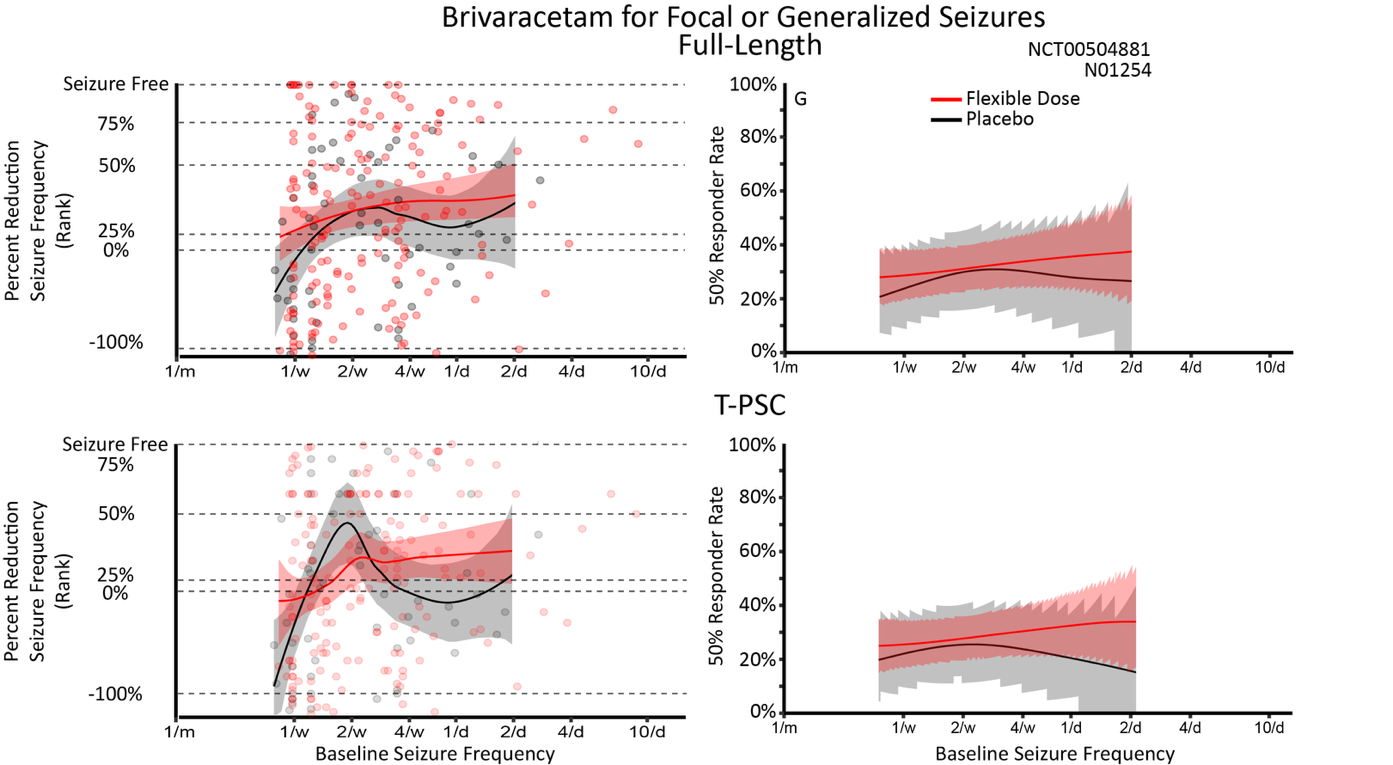


Supplemental Figure 11: Illustration of the individual results from the trial of brivaracetam for focal or generalized seizures (N01254, NCT00504881). No significant shift in either percent reduction or 50% responder rate (parallel slope MPR p=0.19; 50RR p=0.45). Each dot reflects an individual participant. Error bars of percent reduction of seizure frequency reflect 95% confidence intervals of the rank. Error bars of 50% responder rate reflect Clopper-Pearson 95% confidence intervals.


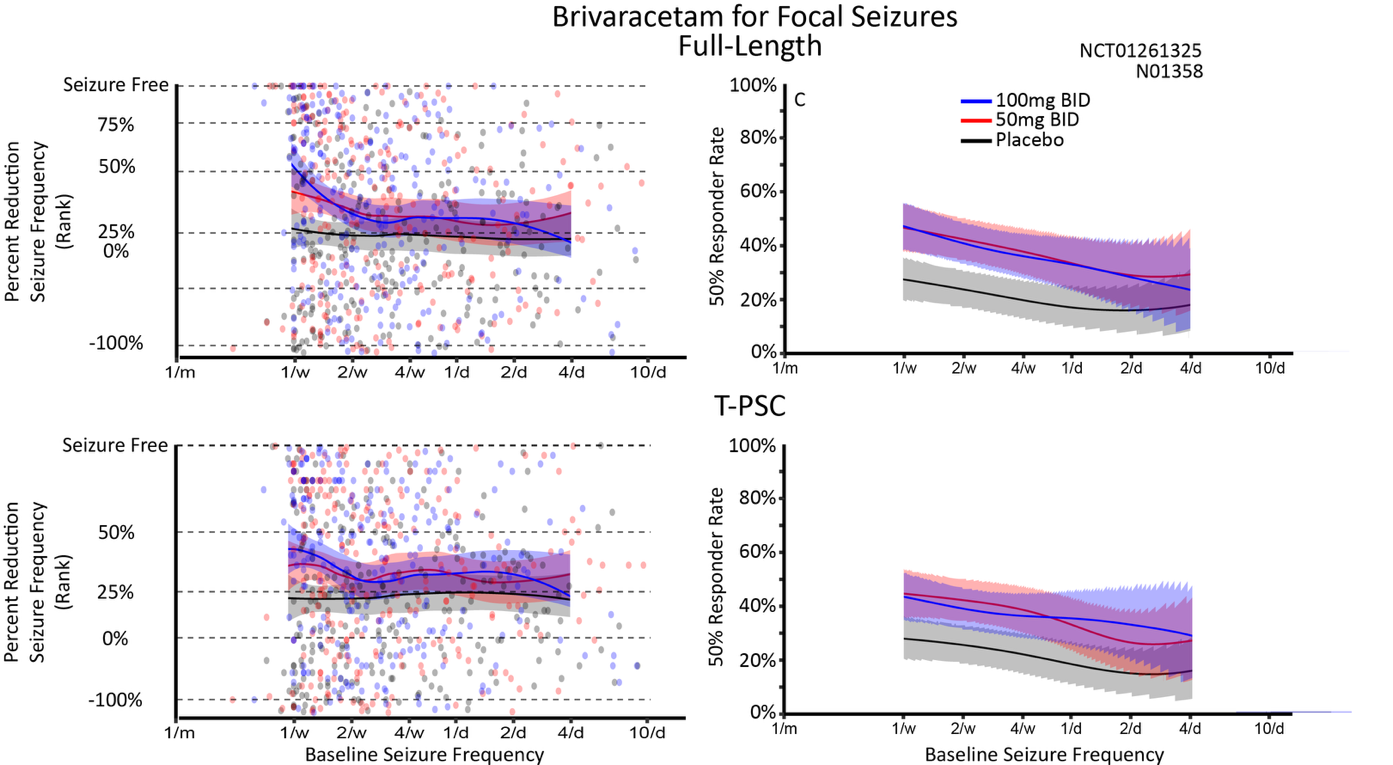


Supplemental Figure 12: Illustration of the individual results from the trial of brivaracetam for focal seizures (N01358, NCT01261325). Parallel shift in increased 50% reduction in people with lower seizure frequency (50RR p=0.15) but no significant parallel shift in percent reduction (MPR p=0.89). Each dot reflects an individual participant. Error bars of percent reduction of seizure frequency reflect 95% confidence intervals of the rank. Error bars of 50% responder rate reflect Clopper-Pearson 95% confidence intervals.


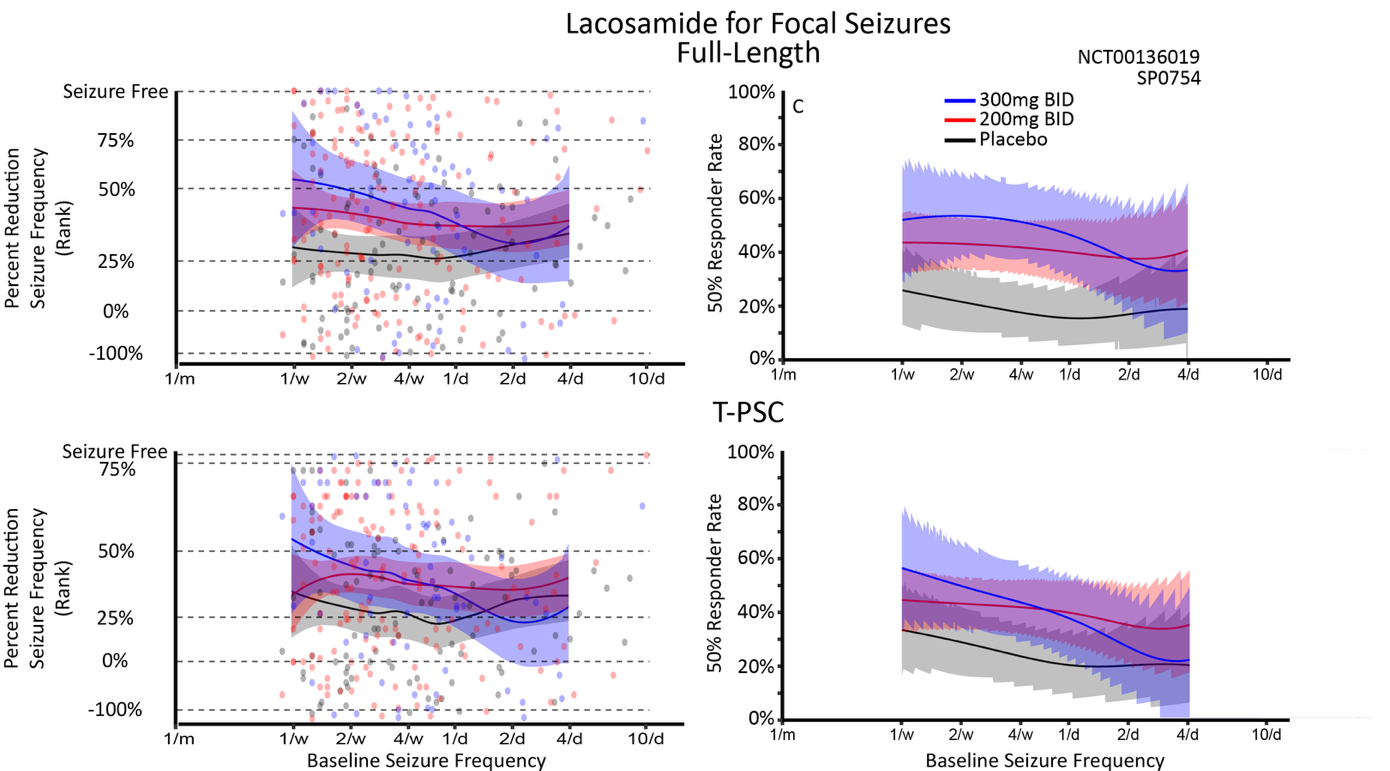


Supplemental Figure 13: Illustration of the individual results from the trial of lacosamide for focal seizures (SP0754, NCT00136019). No significant shift in percent seizure frequency reduction and 50R responder rate (MPR p=0.12; 50RR p=0.94). Each dot reflects an individual participant. Error bars of percent reduction of seizure frequency reflect 95% confidence intervals of the rank. Error bars of 50% responder rate reflect Clopper-Pearson 95% confidence intervals.


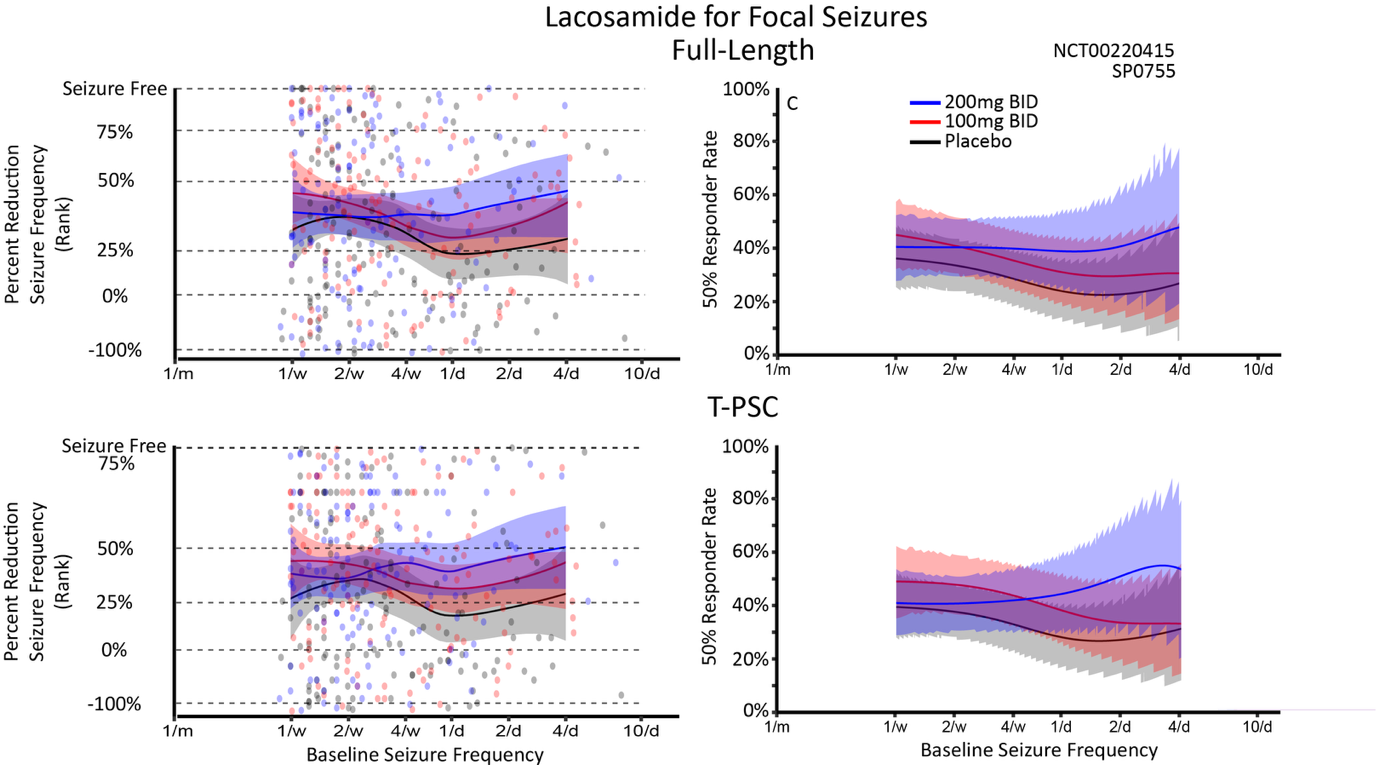


Supplemental Figure 14: Illustration of the individual results from the trial of lacosamide for focal seizures (SP0755, NCT00220415). No significant shift in either percent reduction or 50% responder rate (MPR p=0.13; 50RR p=0.25). Each dot reflects an individual participant. Error bars of percent reduction of seizure frequency reflect 95% confidence intervals of the rank. Error bars of 50% responder rate reflect Clopper-Pearson 95% confidence intervals.
